# Dot1-Dependent Histone H3K79 Methylation Promotes Activation of the Mek1 Meiotic Checkpoint Effector Kinase by Regulating the Hop1 Adaptor

**DOI:** 10.1371/journal.pgen.1003262

**Published:** 2013-01-31

**Authors:** David Ontoso, Isabel Acosta, Fred van Leeuwen, Raimundo Freire, Pedro A. San-Segundo

**Affiliations:** 1 Instituto de Biología Funcional y Genómica, Consejo Superior de Investigaciones Científicas and University of Salamanca, Salamanca, Spain; 2 Division of Gene Regulation, Netherlands Cancer Institute, Amsterdam, The Netherlands; 3 Unidad de Investigación, Hospital Universitario de Canarias, Tenerife, Spain; National Cancer Institute, United States of America

## Abstract

During meiosis, accurate chromosome segregation relies on the proper interaction between homologous chromosomes, including synapsis and recombination. The meiotic recombination checkpoint is a quality control mechanism that monitors those crucial events. In response to defects in synapsis and/or recombination, this checkpoint blocks or delays progression of meiosis, preventing the formation of aberrant gametes. Meiotic recombination occurs in the context of chromatin and histone modifications, which play crucial roles in the maintenance of genomic integrity. Here, we unveil the role of Dot1-dependent histone H3 methylation at lysine 79 (H3K79me) in this meiotic surveillance mechanism. We demonstrate that the meiotic checkpoint function of Dot1 relies on H3K79me because, like the *dot1* deletion, *H3-K79A* or *H3-K79R* mutations suppress the checkpoint-imposed meiotic delay of a synapsis-defective *zip1* mutant. Moreover, by genetically manipulating Dot1 catalytic activity, we find that the status of H3K79me modulates the meiotic checkpoint response. We also define the phosphorylation events involving activation of the meiotic checkpoint effector Mek1 kinase. Dot1 is required for Mek1 autophosphorylation, but not for its Mec1/Tel1-dependent phosphorylation. Dot1-dependent H3K79me also promotes Hop1 activation and its proper distribution along *zip1* meiotic chromosomes, at least in part, by regulating Pch2 localization. Furthermore, *HOP1* overexpression bypasses the Dot1 requirement for checkpoint activation. We propose that chromatin remodeling resulting from unrepaired meiotic DSBs and/or faulty interhomolog interactions allows Dot1-mediated H3K79-me to exclude Pch2 from the chromosomes, thus driving localization of Hop1 along chromosome axes and enabling Mek1 full activation to trigger downstream responses, such as meiotic arrest.

## Author Summary

In sexually reproducing organisms, meiosis divides the number of chromosomes by half to generate gametes. Meiosis involves a series of interactions between maternal and paternal chromosomes leading to the exchange of genetic material by recombination. Completion of these processes is required for accurate distribution of chromosomes to the gametes. Meiotic cells possess quality-control mechanisms (checkpoints) to monitor those critical events. When failures occur, the checkpoint blocks meiotic progression to prevent the formation of aneuploid gametes. Genetic information is packaged into chromatin; histone modifications regulate multiple aspects of DNA metabolism to maintain genomic integrity. Dot1 is a conserved methyltransferase, responsible for histone H3 methylation at lysine 79, that is required for the meiotic recombination checkpoint. Here we decipher the molecular mechanism underlying Dot1 meiotic checkpoint function. We show that Dot1 catalytic activity correlates with the strength of the checkpoint response. By regulating Pch2 chromatin distribution, Dot1 controls localization of the chromosome axial component Hop1, which, in turn, contributes to activation of Mek1, the major effector kinase of the checkpoint. Our findings suggest that, in response to meiotic defects, the chromatin environment created by a constitutive histone mark orchestrates distribution of structural components of the chromosomes supporting activation of the meiotic checkpoint.

## Introduction

During the specialized meiotic cell cycle, two rounds of chromosome segregation follow a single phase of DNA replication dividing the number of chromosomes by half to generate haploid gametes. One of the hallmarks of meiosis is the complex interaction between homologous chromosomes (homologs) involving synapsis and recombination. During meiotic prophase I, homologs find each other, get aligned and finally closely associate along their entire length (synapsis) in the context of the synaptonemal complex (SC). The SC is a tripartite structure composed of two lateral elements (LEs), connected by transverse filaments, which constitute the central region. The chromatin of both sister chromatids of each homolog is organized in loops attached at their base to each of the LEs [1], [2]. In budding yeast, the Red1 and Hop1 proteins localize to the LEs [3], whereas the Zip1 protein is a major component of the SC central region [4], [5]. Concomitant with SC development, meiotic recombination takes place. Meiotic recombination initiates with programmed double-strand breaks (DSBs) introduced by Spo11 and accessory proteins [6]. Meiotic DSBs are preferentially repaired using an intact non-sister chromatid resulting in physical connections between homologs (chiasmata), which promote proper chromosome segregation.

Accurate distribution of chromosomes to the progeny is essential for generation of functional gametes; thus, meiotic cells are endowed with a meiosis-specific surveillance mechanism, the so-called pachytene checkpoint or meiotic recombination checkpoint, which contributes to faithful chromosome segregation. In response to defects in meiotic recombination and/or chromosome synapsis, the pachytene checkpoint is triggered and blocks or delays exit from prophase of meiosis I to prevent aberrant chromosome segregation and the formation of aneuploid meiotic products [7], [8].

This evolutionary-conserved quality-control mechanism operates from yeast to mammals. In *S. cerevisiae*, the meiotic recombination checkpoint responding to unrepaired resected DSBs shares the same sensors with the DNA damage checkpoint operating in vegetative cells, including the Mec1/Ddc2 kinase, Rad24 and the 9-1-1 complex [9]–[11]. However, the Rad9 adaptor and the Rad53 checkpoint kinase are dispensable for this meiotic checkpoint. On the contrary, given the special chromosomal context where meiotic recombination takes place, the meiosis-specific axial chromosomal components Red1 and Hop1 act as adaptors between the upstream sensors and the downstream Mek1 meiotic effector kinase, which, like Rad53, is hyperphosphorylated upon checkpoint activation [12], [13]. In turn, the meiotic cell cycle delay is imposed by inhibition of crucial regulators of meiosis I progression, including the cyclin-dependent kinase Cdc28, the transcription factor Ndt80 and the polo-like kinase Cdc5 [14]–[19]. Budding yeast meiotic mutants, such as *zip1* (defective in SC and crossover formation) or *dmc1* (defective in the strand invasion step of interhomolog recombination), are invaluable genetic tools to activate the meiotic recombination checkpoint.

Meiotic recombination as well as detection and signaling of recombination intermediates by the checkpoint machinery occur in the context of chromatin; therefore, histone posttranslational modifications are expected to play important roles on these processes [20]. For example, Set1-dependent H3K4 methylation is linked to meiotic DSB formation [21], [22]. On the other hand, the Dot1 histone methyltranferase, which targets H3K79 [23]–[25], is largely dispensable for unperturbed meiosis, but is essential for meiotic checkpoint function. Mutation of *DOT1* relieves the meiotic prophase arrest of *zip1* and *dmc1* mutants resulting in defective meiotic products [26]. Dot1 is also involved in several aspects of the DNA damage response in vegetative yeast cells and the DOT1L mammalian homolog also plays crucial cellular functions [27]. However, the molecular mechanisms underlying the meiotic checkpoint function of Dot1 are unknown.

Here, we investigated the role of Dot1-dependendent H3K79 methylation in *zip1*-induced checkpoint activation. By manipulation of Dot1 catalytic activity and levels, we found that the extent of H3K79 trimethylation correlates with the strength of checkpoint-imposed meiotic delay. We demonstrate that while the meiotic defects of a synapsis and recombination-deficient *zip1* mutant are correctly sensed by Mec1-Ddc2 in the absence of H3K79me, activation of the downstream effector kinase Mek1 is impaired. We dissected the Mek1 phosphorylation events and found that Dot1 promotes its Hop1-dependent dimerization and auto-phosphorylation. Finally, we show that the effect of Dot1-dependent H3K79me on Hop1 localization is exerted, at least in part, by excluding Pch2 from the chromosomes. Our results indicate that constitutive methylation of H3K79 by Dot1 is required for proper chromosomal recruitment of Hop1 to relay the checkpoint signal to Mek1 in response to meiotic defects.

## Results

### Histone H3K79 methylation regulates the meiotic recombination checkpoint

**Figure 1:**
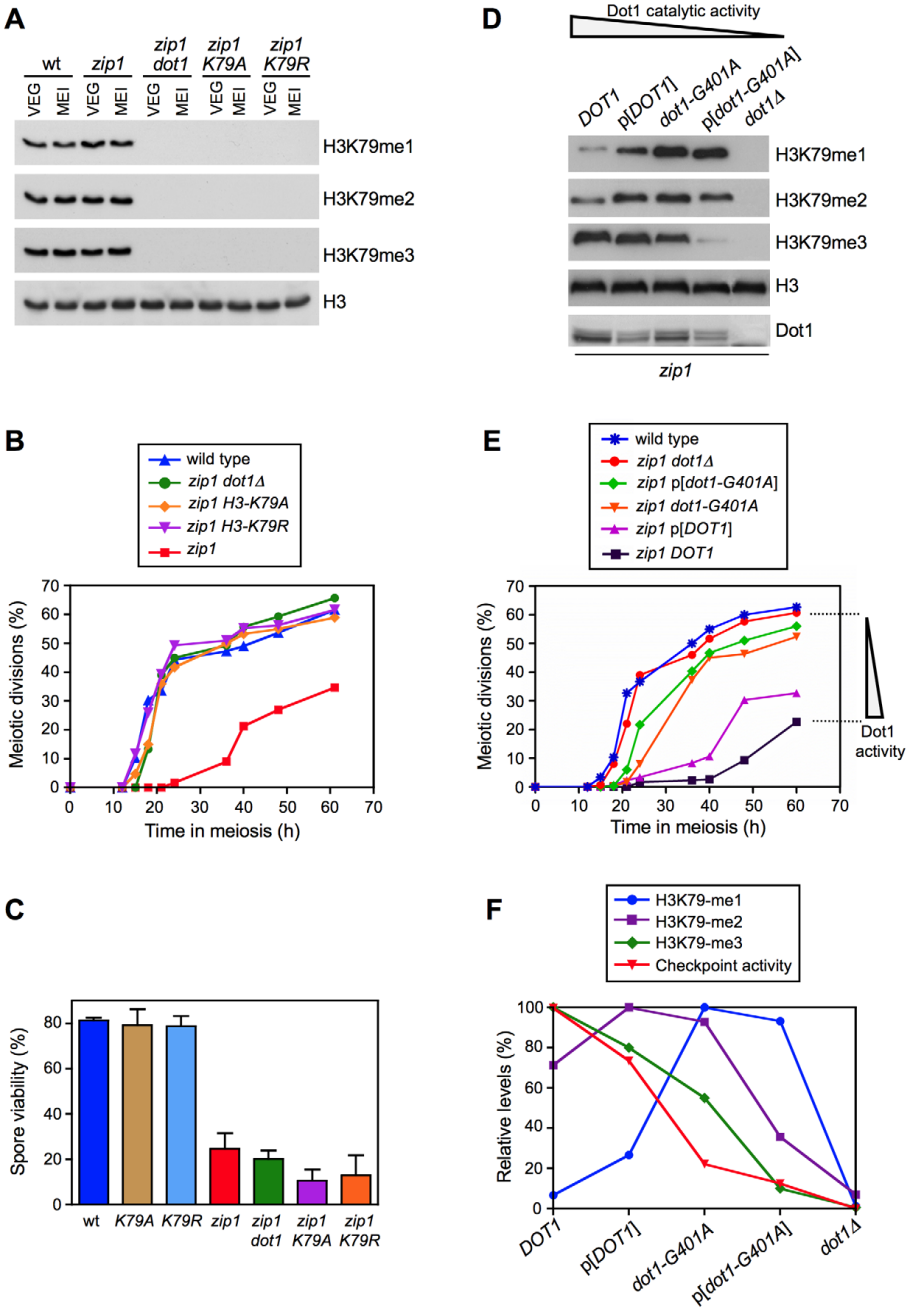
Methylation of H3K79 by Dot1 is essential for meiotic recombination checkpoint function. (A) Western blot analysis of H3K79 methylation in vegetative (VEG) and meiotic (MEI) cells to compare H3K79 methylation levels in different mutant backgrounds. Samples of meiotic cells were taken 15 h after meiosis induction. Total histone H3 is shown as a loading control. (B) Suppression of *zip1* meiotic delay by *dot1* or by *H3-K79A* and *H3-K79R* mutations. Time course of meiotic nuclear divisions; the percentage of cells containing more than two nuclei is represented. (C) Spore viability determined by tetrad dissection. At least 240 spores were scored for each strain. Means and standard deviations are shown. Strains for (A), (B) and (C) are: DP806 (wild type), DP807 (*H3-K79A*), DP808 (*H3-K79R*), DP809 (*zip1*), DP812 (*zip1 dot1*), DP810 (*zip1 H3-K79A*) and DP811 (*zip1 H3-K79R*). (D) Western blot analysis of H3K79 methylation in *zip1* strains producing different versions of Dot1 either from the endogenous loci (*DOT1* and *dot1-G401A*) or from a centromeric plasmid (p[*DOT1*] and p[*dot1-G401A*]). Samples were taken 24 h after meiosis induction. Dot1 levels are also shown. Total histone H3 serves as a loading control. (E) Time course of meiotic nuclear divisions; the percentage of cells containing more than two nuclei is represented. (F) Quantification of the relative levels of H3K79 mono-, di-, and tri-methylation. The maximum value of each methylation state was considered 100%. Checkpoint activity represents the ability to impose the *zip1* meiotic delay according to data in (E). The meiotic nuclear division values for the latest time point (60 h) were considered in the calculations. Maximum checkpoint activity (100%) was assigned to the *zip1* strain expressing endogenous wild-type *DOT1*. Strains for (D), (E) and (F) are: DP421 + pRS315 (wild type), DP555 + pRS315 (*zip1 dot1Δ*), DP555 + pRS315-DOT1 (*zip1* p[*DOT1*]), DP555 + pFvL54 (*zip1* p[*dot1-G401A*]), DP556 + pRS315 (*zip1 DOT1*) and DP560 + pRS315 (*zip1 dot1-G401A*).

Dot1 catalyzes the mono-, di- and tri-methylation of histone H3K79 (Figure 1A) by a non-processive mechanism [27], [28] and plays a crucial role in the meiotic recombination checkpoint [26]. Notably, we found that overall levels of H3K79me do not significantly change upon meiosis induction (Figure 1A, compare vegetative and meiotic cells; Figure S1A) or upon meiotic checkpoint activation (Figure 1A, compare wild type and *zip1* meiotic cells; Figure S1A). Moreover, H3K79me meiotic levels were not significantly altered in the *spo11* mutant, lacking meiotic recombination [6], or in other mutants defective in the meiotic recombination checkpoint, such as *rad24*, *pch2* and *ddc2* [10], [11], [29] (Figure S1B). Therefore, to determine whether regulation of the meiotic recombination checkpoint by Dot1 relies on H3K79me, we generated and analyzed *H3-K79R* and *H3-K79A* mutants, in which the lysine 79 targeted by Dot1 cannot be methylated (Figure 1A). Importantly, like *dot1*, both methylation-site mutants suppressed the pronounced checkpoint-imposed meiotic delay of the *zip1* mutant (Figure 1B). In an otherwise wild-type background, *DOT1* deletion has no or little meiotic effects and spore viability is high [26], [30]; likewise, the *H3-K79R* and *H3-K79A* single mutants showed wild-type levels of spore viability (Figure 1C), suggesting that H3K79me is dispensable in unperturbed meiosis. However, similar to *zip1 dot1*, spore viability was strongly reduced in *zip1 H3-K79R* and *zip1 H3-K79A* (Figure 1C), indicating that the defects conferred by *zip1* persist in the double mutants despite their wild-type kinetics of meiotic progression. Thus, Dot1-dependent H3K79me is essential for meiotic recombination checkpoint function.

To further investigate the regulation of the meiotic checkpoint by H3K79me, we monitored checkpoint function in *zip1* diploid strains exhibiting gradually decreased Dot1 activity. In order to generate this set of strains, we used the combination of the *dot1-G401A* allele, which confers partial catalytic activity [28], with the expression of *DOT1* (or *dot1-G401A*) from a plasmid, which results in lower protein levels (Figure 1D; [31]). Analysis of H3K79-me1, -me2 and -me3 levels in meiotic cells confirmed a gradually reduced Dot1 activity following this order: *DOT1*>p[*DOT1*]>*dot1-G401A*>p[*dot1-G401A*]>*dot1Δ*, as manifested by progressively reduced H3K79-me3 and, conversely, progressively increased H3K79-me1 (Figure 1D). Interestingly, meiotic checkpoint activity, monitored as the ability to impose the *zip1* meiotic delay, also showed a gradual decrease mirroring the drop in Dot1 catalytic function (Figure 1E). Quantification of the relative levels of each H3K79 methylation state revealed a marked correlation between H3K79-me3 and checkpoint function (Figure 1F). Thus, the status of H3K79 methylation modulates the meiotic recombination checkpoint, with the H3K79-me3 form being the most relevant to sustain the checkpoint response.

### Dot1 is required for activation of the Mek1 effector kinase

**Figure 2:**
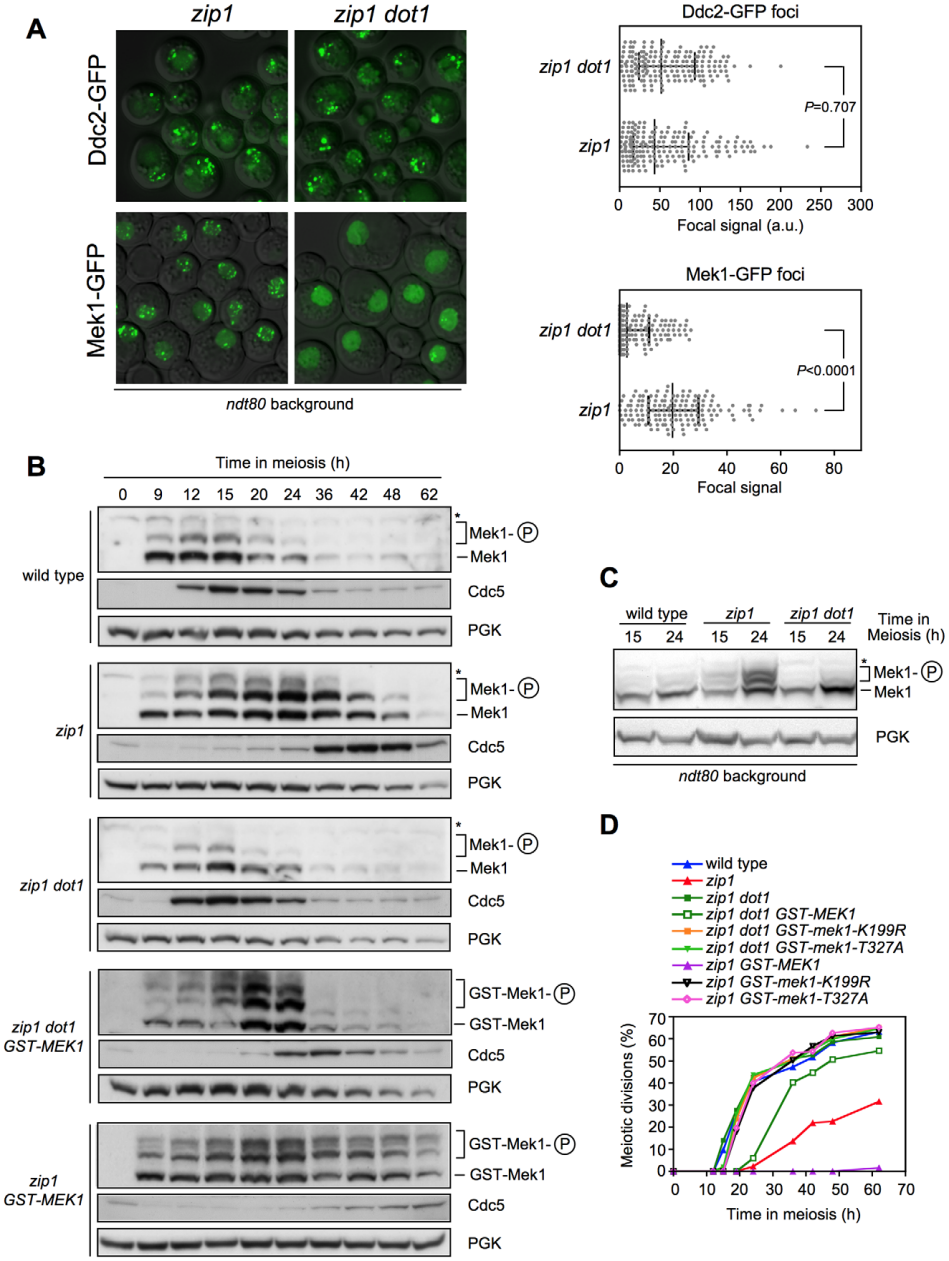
Dot1 is required for checkpoint-promoted localization and activation of Mek1. (A) Formation of *zip1*-induced Mek1 foci is defective in the absence of Dot1. Representative images of Ddc2-GFP and Mek1-GFP foci in *zip1* and *zip1 dot1* cells after 24 h in meiosis. Strains are DP460 (*zip1 DDC2-GFP*), DP579 (*zip1 dot1 DDC2-GFP*), DP582 (*zip1 MEK1-GFP*) and DP583 (*zip1 dot1 MEK1-GFP*). All strains are *ndt80*-arrested at pachytene. The graphs show the quantification of Ddc2 and Mek1 foci formation from the same samples determined as the intensity of the total focal GFP signal relative to total nuclear signal (a.u., arbitrary units). Error bars represent the median with interquartile range. Each spot in the plot represents the foci intensity of every nucleus measured. 175 and 150 nuclei were analyzed for Ddc2-GFP and Mek1-GFP, respectively. (B) Western blot analysis of Mek1 activation by phosphorylation and Cdc5 production throughout meiosis in wild type (DP421), *zip1* (DP422), *zip1 dot1* (DP555), *zip1 dot1 GST-MEK1* (DP785) and *zip1 GST-MEK1* (DP792) using Phos-tag gels. PGK was used as a loading control. (C) Analysis of Mek1 activation in *ndt80*-arrested cells. Strains are DP424 (wild type), DP428 (*zip1*) and DP655 (*zip1 dot1*). (D) Time course of meiotic nuclear divisions; the percentage of cells containing more than two nuclei is represented. Strains are: DP421 (wild type), DP422 (*zip1*), DP555 (*zip1 dot1*), DP785 (*zip1 dot1 GST-MEK1*), DP783 (*zip1 dot1 GST-mek1-K199R*), DP784 (*zip1 dot1 GST-mek1-T327A*), DP792 (*zip1 GST-MEK1*), DP790 (*zip1 GST-mek1-K199R*) and DP791 (*zip1 GST-mek1-T327A*).

Next, we sought to determine where in the meiotic recombination checkpoint pathway Dot1-dependent H3K79me is acting. We first analyzed checkpoint sensor function by monitoring the formation of *zip1*-induced Ddc2-GFP foci [11]. Formation of Ddc2 foci was not disrupted in the absence of Dot1 (Figure 2A), suggesting that H3K79me is not required for the ability of Mec1-Ddc2 to detect meiotic recombination intermediates. Upon checkpoint activation, the Mek1 effector kinase forms nuclear foci that can be detected both on chromosome spreads ([9]; see below) and in live meiotic cells (Figure 2A). Strikingly, we found that the *zip1* mutant accumulated multiple discrete Mek1-GFP foci during meiotic prophase, whereas most *zip1 dot1* cells displayed a diffuse Mek1 nuclear signal and only occasional foci were observed (Figure 2A) indicating that Dot1 promotes checkpoint-induced association of Mek1 to meiotic chromosomes (see below).

Mek1 is activated by phosphorylation in mutants that trigger the meiotic recombination checkpoint, including *zip1* [12], [14], [32], [33]; therefore, we followed Mek1 phosphorylation throughout meiosis in wild-type, *zip1* and *zip1 dot1* cells using Phos-tag gels (Figure 2B). In the wild type, Mek1 was weakly and transiently activated during the peak of meiotic prophase in this strain background (around 12–15 h). In contrast, Mek1 was hyperactivated in *zip1* cells as evidenced by the presence of additional, more persistent, and stronger phosphorylated forms. However, Mek1 hyperactivation was not observed in the *zip1 dot1* double mutant; like in wild type, only a weak and transient phosphorylated form was detected. To rule out the possibility that the difference between *zip1* and *zip1 dot1* were due to their different kinetics of meiotic progression (*zip1* exhibits a marked delay that is bypassed in *zip1 dot1*; Figure 1B), we monitored Mek1 phosphorylation in *ndt80* pachytene-arrested cells. As presented in Figure 2C, *zip1*-induced hyperphosphorylation of Mek1 was severely impaired in the absence of Dot1.

In summary, these results place Dot1 function upstream of Mek1 in the meiotic recombination checkpoint pathway and indicate that, whereas Mec1/Ddc2 act independently of H3K79 methylation to sense meiotic defects, Dot1 is required for checkpoint-induced activation of Mek1.

### Autophosphorylation of Mek1 depends on Dot1

**Figure 3:**
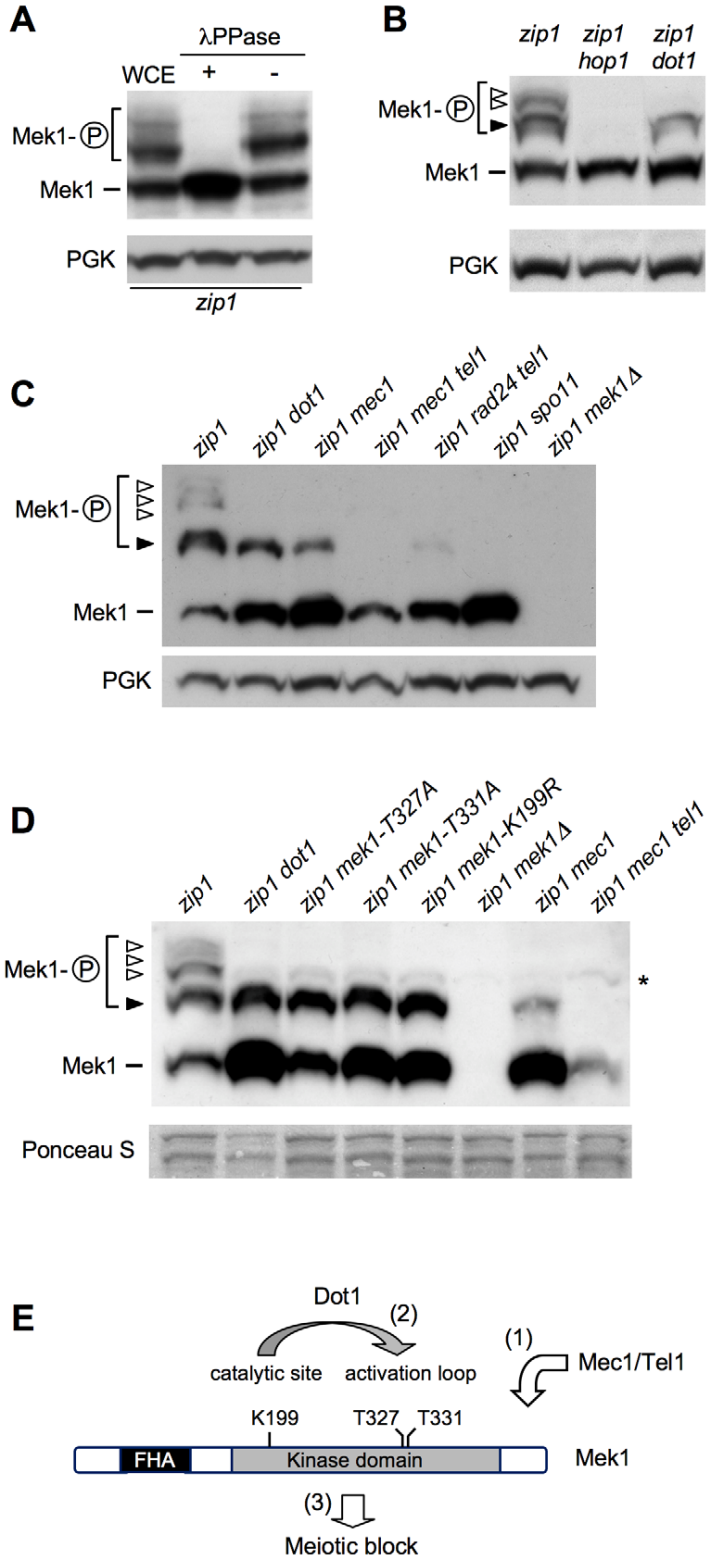
Dot1 contributes to Mek1 activation by autophosphorylation. (A) Whole cell extracts (WCE) from a *zip1 ndt80* culture at 24 h in meiosis were incubated in the presence (+) or absence (−) of lambda phosphatase (λPPase). (B), (C) and (D) Detection of different phosphorylated forms of Mek1 in *ndt80*-arrested cells after 24 h in meiosis using high-resolution Phos-tag gels. Basal Mek1 (line) and several phosphorylated forms (black and white arrowheads) are indicated; see text for explanation. PGK or Ponceau S staining were used as loading controls. Asterisk in (D) marks a weak non-specific band. (E) Schematic representation of a model for the sequential phosphorylation events leading to Mek1 activation and the relevant mutations analyzed above. (1) Priming phosphorylation by Mec1/Tel1 (black arrowhead in B, C, D) is followed by (2) autophosphorylation of Mek1 (white arrowheads in B, C, D) leading to its full activation and (3) the checkpoint response. H3K79 methylation by Dot1 contributes to Mek1 autophosphorylation. Strains were: (A); DP428 (*zip1*). (B); DP428 (*zip1*), DP701 (*zip1 hop1*) and DP655 (*zip1 dot1*). (C); DP428 (*zip1*), DP655 (*zip1 dot1*), DP680 (*zip1 mec1*), DP861 (*zip1 mec1 tel1*), DP877 (*zip1 rad24 tel1*), DP728 (*zip1 spo11*) and DP674 (*zip1 mek1Δ*). (D); DP885 (*zip1*), DP890 (*zip1 dot1*), DP886 (*zip1 mek1-T327A*), DP887 (*zip1 mek1-T331A*), DP888 (*zip1 mek1-K199R*), DP674 (*zip1 mek1Δ*), DP680 (*zip1 mec1*) and DP861 (*zip1 mec1 tel1*).

In *ndt80*-arrested cells, using high-resolution Phos-tag gels, we were able to resolve several *zip1*-induced shifted forms of Mek1 above the basal band (Figure 3A–3D). Phosphatase treatment eliminated all band shifts indicating that they represent distinct phosphorylated forms (Figure 3A). We used different *mek1* versions carrying specific mutations, as well as mutants in upstream components of the checkpoint pathway, in order to determine the contribution of different phosphorylation events to the observed checkpoint-induced Mek1 forms in *zip1 ndt80* cells (Figure 3E). Mek1 phosphorylation was completely abolished in the *hop1* mutant, lacking a LE-component meiotic checkpoint adaptor [3], [34], [35] (Figure 3B) and in the *spo11* mutant, which does not initiate recombination [36] (Figure 3C). However, in the absence of Dot1, only the upper phosphorylated bands were eliminated (Figure 3B–3D, white arrowheads), but the form immediately above the basal Mek1 band remained intact (Figure 3B–3D, black arrowhead). Interestingly, this moderately-shifted form was reduced in *mec1* cells and virtually disappeared in *mec1 tel1* and *rad24 tel1* mutants (Figure 3C and 3D, black arrowhead), suggesting that it arises from Mec1/Tel1-dependent phosphorylation. On the other hand, the kinase-dead *mek1-K199R* allele, as well as the autophosphorylation-defective *mek1-T327A* and *mek1-T331A* mutants [33], specifically lacked the upper bands displaying the stronger mobility shift, suggesting that they result from Mek1 autophosphorylation (Figure 3D, white arrowheads). In contrast, the Mek1 form immediately above the basal band (i.e., resulting from Mec1/Tel1 action) remained invariable in those *mek1* mutants (Figure 3D, black arrowhead). Thus, interestingly, the *zip1 dot1* mutant showed a similar pattern to that of *zip1 mek1-K199R*, *zip1 mek1-T327A* or *zip1 mek1-T331A* (Figure 3D), strongly suggesting that Dot1 is mainly required for Mek1 autophosphorylation, but not for its Mec1/Tel1-dependent phosphorylation (Figure 3E).

It has been proposed that dimerization of Mek1 promotes its function, likely by facilitating *in trans* autophosphorylation [33], [37]. Thus, we hypothesized that Dot1 could be required for Mek1 dimerization. Importantly, we found that GST-driven forced dimerization of Mek1 restored its full phosphorylation even in the absence of Dot1, although Mek1 activation was not maintained at late time points (Figure 2B). Consistently, expression of *GST-MEK1* in *zip1 dot1* strains conferred a brief, but significant, meiotic delay (Figure 2D). As previously reported, the *zip1 GST-MEK1* mutant was completely halted (Figure 2D) [37], and we found that this block was accompanied by the persistent hyperphosphorylation of GST-Mek1 (Figure 2B). The permanent or transient arrest conferred by GST-Mek1 in *zip1* or *zip1 dot1*, respectively, was completely relieved when inactive kinase (*GST-mek1-K199R*) or autophosphorylation-defective (*GST-mek1-T327A*) versions were introduced (Figure 2D), confirming that in *GST-MEK1* strains, meiotic progression was slowed down by forced Mek1 activation and not by another unrelated cause. To further support this conclusion, we monitored another downstream molecular marker of pachytene checkpoint activation, such as the inhibition of the production of the Cdc5 polo-like kinase [14], [18]. As expected, whereas induction of Cdc5 was delayed in *zip1* cells, the *zip1 dot1* double mutant displayed wild-type kinetics of Cdc5 production (Figure 2B). Strikingly, consistent with the kinetics of meiotic progression (Figure 2D), expression of *GST-MEK1* in *zip1 dot1* cells restored a significant delay in Cdc5 induction. Furthermore, Cdc5 production was severely impaired in the arrested *zip1 GST-MEK1* strain (Figure 2B). In summary, these observations indicate that artificial dimerization of Mek1 partially overcomes Dot1 requirement for Mek1 activation and further supports the conclusion that Dot1 function promotes Mek1 autophosphorylation.

### Dot1 is required for localization and activation of the Hop1 meiotic checkpoint adaptor

**Figure 4:**
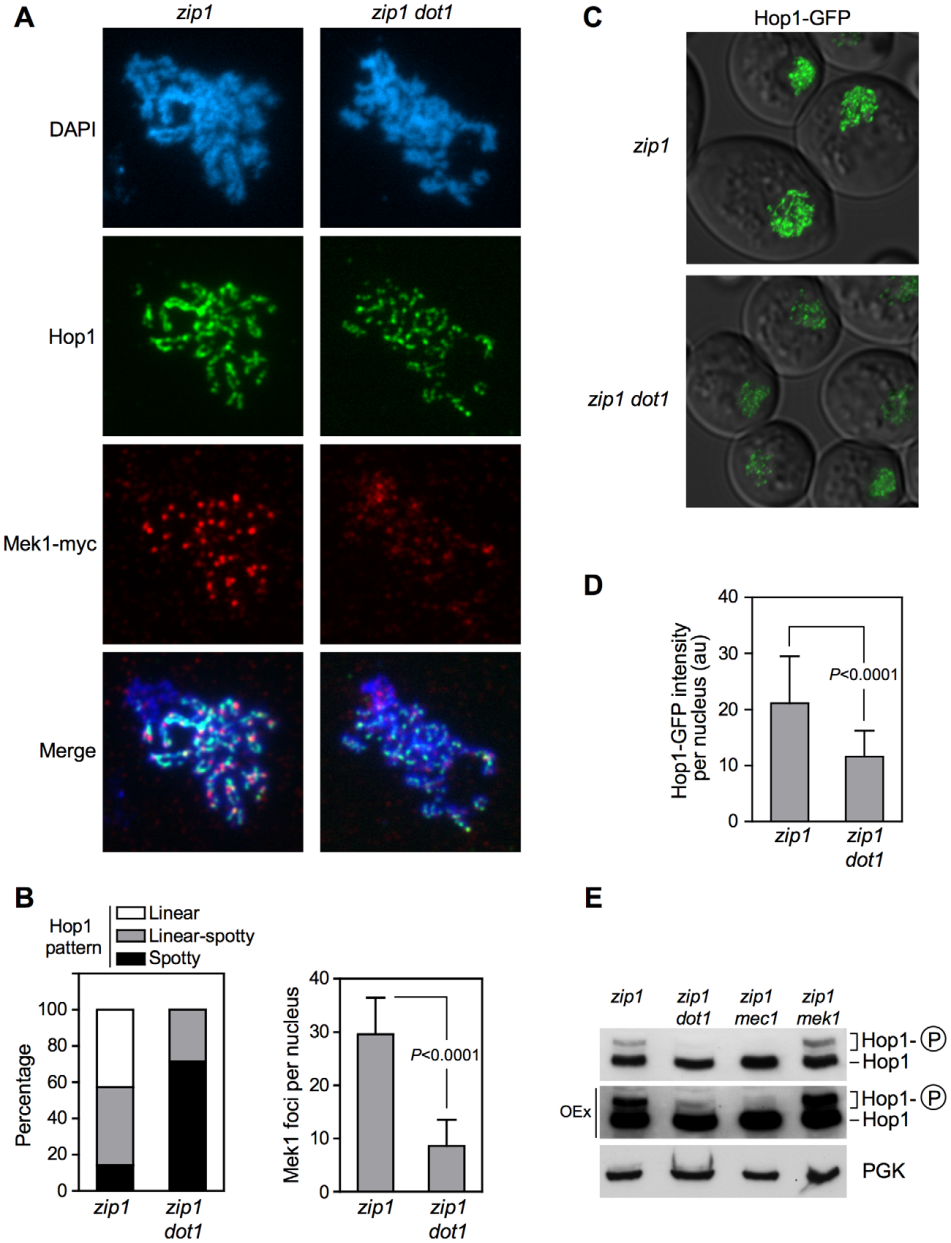
Dot1 is required for *zip1*-induced localization and activation of the Hop1 meiotic checkpoint adaptor. (A) Immunofluorescence of meiotic chromosome spreads stained with DAPI (blue), anti-Hop1 (green) and anti-myc (red) antibodies. Representative nuclei are shown. The same exposure time was used to capture the signal from the different strains. Spreads were prepared 24 h after meiotic induction of *ndt80* cells. Strains are: DP848 (*zip1*) and DP849 (*zip1 dot1*). (B) Quantification of the Hop1 staining pattern (left) and the number of Mek1 foci (right) on spread chromosomes analyzed as in (A). 14 and 21 nuclei were scored for *zip1* and *zip1 dot1*, respectively. (C) Representative images of *ndt80*-arrested cells expressing *HOP1-GFP* in *zip1* (DP964) and *zip1 dot1* (DP965) captured after 24 h in meiosis. (D) Quantification of the Hop1-GFP signal intensity on fluorescence images (a.u., arbitrary units). 300 individual nuclei were analyzed for each strain. (E) Dot1 is required for Hop1 phosphorylation. Western blot analysis of Hop1 in cell extracts obtained 24 h after meiotic induction in *ndt80* cells. The middle panel corresponds to an overexposure (OEx) of the blot shown in the upper panel. PGK was used as a loading control. Strains are: DP428 (*zip1*), DP655 (*zip1 dot1*), DP680 (*zip1 mec1*) and DP674 (*zip1 mek1*). Means, standard deviations and *P-*values are shown in (B) and (D).

**Figure 7:**
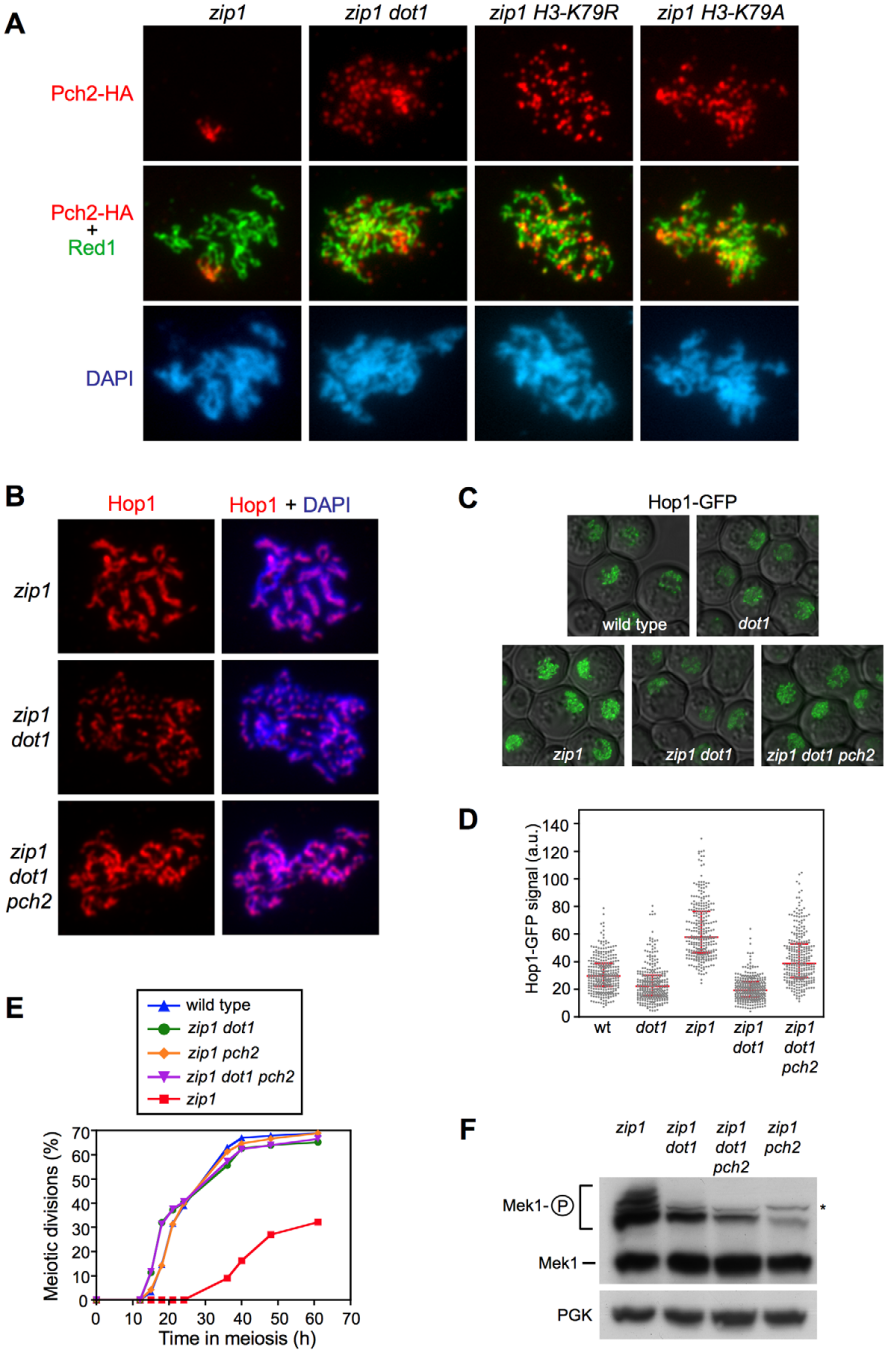
H3K79me controls Hop1 localization by excluding Pch2 from chromosomes. (A) H3K79me is required to prevent Pch2 localization outside of the rDNA. Immunofluorescence of meiotic chromosome spreads stained with DAPI (blue), anti-HA (red) and anti-Red1 (green) antibodies. Strains are: DP1050 (*zip1*), DP1053 (*zip1 dot1*), DP1052 (*zip1 H3-K79R*) and DP1051 (*zip1 H3-K79A*). (B–D) The absence of Pch2 partially restores Hop1 chromosomal abundance in *zip1 dot1*. (B) Immunofluorescence of meiotic chromosome spreads stained with DAPI (blue) and anti-Hop1 antibody (red). Strains are: DP428 (*zip1*), DP655 (*zip1 dot1*) and DP1054 (*zip1 dot1 pch2*). (C) Representative images of cells expressing *HOP1-GFP* in wild type (DP963), *dot1* (DP966), *zip1* (DP964), *zip1 dot1* (DP965) and *zip1 dot1 pch2* (DP1027). (D) Quantification of the Hop1-GFP signal intensity on fluorescence images (a.u., arbitrary units). 300 individual nuclei were analyzed for each strain. Each spot in the plot represents the fluorescence intensity of every nucleus measured. Error bars represent the median with interquartile range. *P*<0.01 in pairwise comparisons. In all cases (A–C), spreads were prepared and GFP images were taken 24 h after meiotic induction in *ndt80* strains. (E, F) The absence of Pch2 does not restore the pachytene checkpoint response in *zip1 dot1*. (E) Time course of meiotic nuclear divisions; the percentage of cells containing more than two nuclei is represented. Strains are: DP421 (wild type), DP422 (*zip1*), DP555 (*zip1 dot1*), DP1029 (*zip1 pch2*) and DP1041 (*zip1 dot1 pch2*). (F) Western blot analysis of *zip1*-induced Mek1 phosphorylation in *ndt80* strains. PGK was used as a loading control. The asterisk marks a presumed non-specific band (see Figure 3D). Strains are: DP428 (*zip1*), DP655 (*zip1 dot1*), DP881 (*zip1 pch2*) and DP1054 (*zip1 dot1 pch2*).

It has been reported that activated Hop1 promotes Mek1 dimerization via a C-terminal domain [33], [38]; therefore, we investigated whether the effect of Dot1 on Mek1 phosphorylation was mediated by Hop1. First, we studied Hop1 localization on chromosome spreads of *ndt80*-arrested *zip1* and *zip1 dot1* strains. As previously described [3], Hop1 displayed a predominantly linear staining along the lateral elements of *zip1* chromosomes. In contrast, only short stretches of Hop1 could be detected in the *zip1 dot1* mutant, which showed a predominating Hop1 punctate pattern (Figure 4A and 4B, left panel). Consistent with our observations in live cells (Figure 2A), we also detected a marked reduction of Mek1 chromosomal foci in *zip1 dot1*, compared to the *zip1* single mutant (Figure 4A and 4B, right panel). In addition, we also analyzed Hop1 localization in *zip1* and *zip1 dot1* live meiotic cells expressing *HOP1-GFP*. In line with the aberrant distribution on spreads, we observed that Hop1-GFP signal was weaker and less continuous in *zip1 dot1* cells. (Figure 4C and 4D; Video S1). This discontinuous localization of Hop1 does not result from a pronounced alteration of overall chromosome structure, because the SC lateral component Red1 [3] displayed a linear distribution in both *zip1* and *zip1 dot1* strains (Figure S2). On the other hand, the *dot1* single mutant only showed a modest decrease of Hop1-GFP signal compared with the wild type (see Figure 7C and 7D below). Thus, upon *zip1*-induced checkpoint activation, Dot1 enables proper loading or maintenance of Hop1 onto chromosomes.

Since Mec1/Tel1-dependent phosphorylation of Hop1 at defined S/T-Q motifs is required for Mek1 activation and localization [34], we examined *zip1*-induced Hop1 phosphorylation in the absence of Dot1, by monitoring its gel mobility shift. As shown in Figure 4E, the *zip1 dot1* mutant displayed a severe defect in Hop1 phosphorylation, similar to the *zip1 mec1* and *zip1 spo11* mutants also analyzed as controls (Figure 4E and Figure S3). Even after long overexposure of the gels, only a barely visible phosphorylated form of Hop1 could be detected in the absence of Dot1 (Figure 4E).

**Figure 5:**
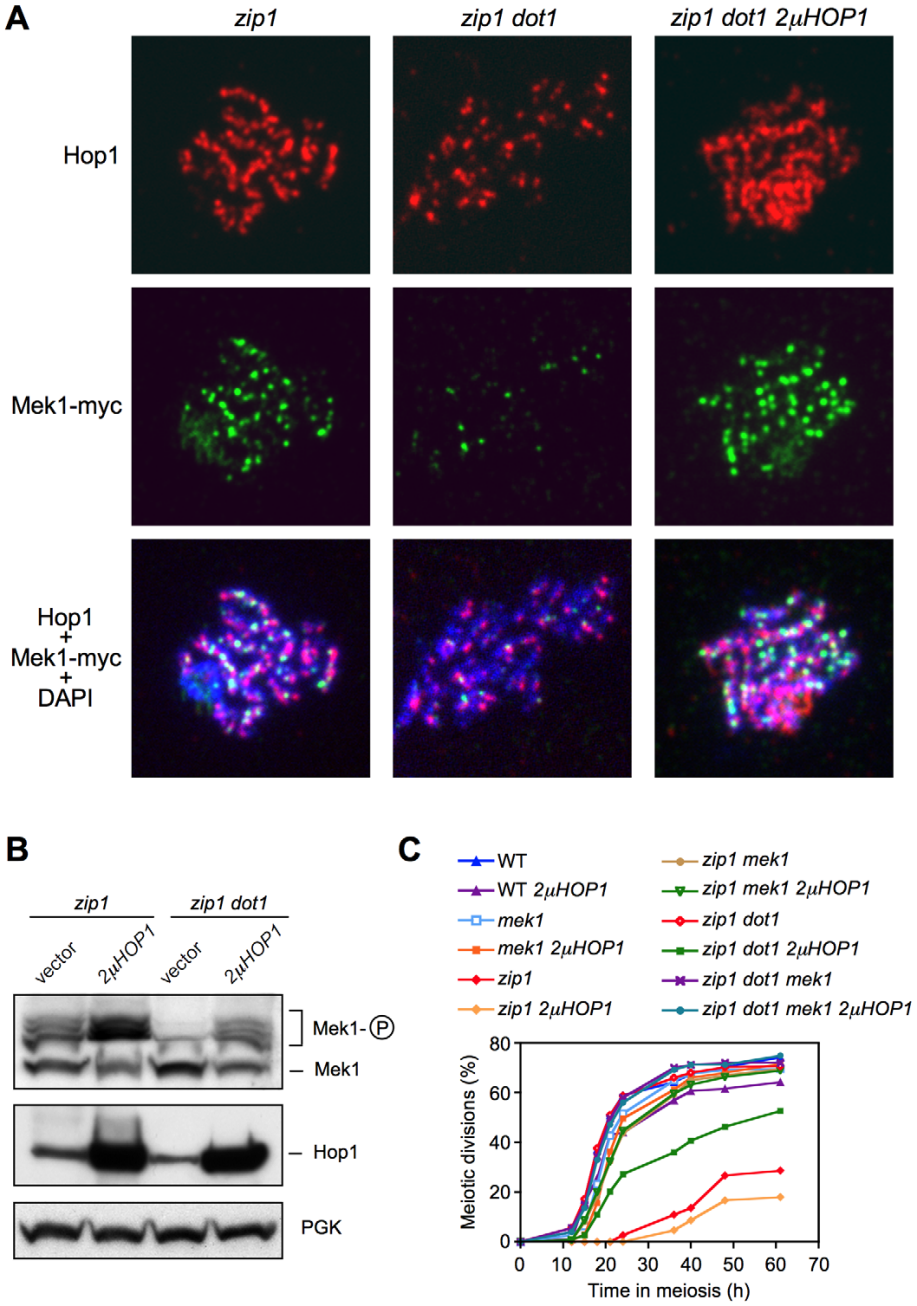
Hop1 overproduction restores Mek1 function in the absence of Dot1. (A) Immunofluorescence of meiotic chromosome spreads stained with DAPI (blue), anti-Hop1 (red) and anti-myc (green) antibodies. Spreads were prepared 24 h after meiotic induction of *ndt80* cells. Strains are: DP848 (*zip1*) and DP884 (*zip1 dot1*) transformed either with empty vector or with a *HOP1* high-copy plasmid (*2μHOP1*). (B) Western blot analysis of Mek1 phosphorylation and Hop1 production in *ndt80* cells after 24 h in meiosis. PGK was a loading control. Strains are DP428 (*zip1*) and DP655 (*zip1 dot1*) transformed either with empty vector or with *2μHOP1*. (C) Time course of meiotic nuclear divisions; the percentage of cells containing more than two nuclei is represented. Strains are DP421 (wild type), DP713 (*mek1*), DP422 (*zip1*), DP714 (*zip1 mek1*), DP555 (*zip1 dot1*) and DP716 (*zip1 dot1 mek1*), transformed either with empty vector or with *2μHOP1*.

These observations suggest that the defect in Mek1 autophosphorylation observed in the absence of Dot1 stems from impaired Hop1 function. To confirm this notion, we overexpressed *HOP1* from a high-copy plasmid in *zip1 dot1* cells. As shown in Figure 5, whereas the *zip1 dot1* mutant transformed with empty vector showed defective Mek1 localization and activation, *HOP1* overexpression in *zip1 dot1* restored Mek1 chromosomal foci (Figure 5A), Mek1 phosphorylation (Figure 5B), and reestablished a substantial meiotic delay (Figure 5C). We found that Hop1 overproduction also conferred a slight reduction in the efficiency of meiotic progression in the wild type (Figure 5C) and further enhanced the *zip1* meiotic delay, as expected from the strong hyperphosphorylation of Mek1 (Figure 5B and 5C). Notably, in all cases (wild type, *zip1* or *zip1 dot1*), the further delay in meiotic progression imposed by high levels of Hop1 was suppressed by the absence of Mek1 (Figure 5C), proving that it was caused from amplified pachytene checkpoint signaling and not from an unrelated cause.

### H3K79me is required for Mek1 and Hop1 phosphorylation and localization

**Figure 6:**
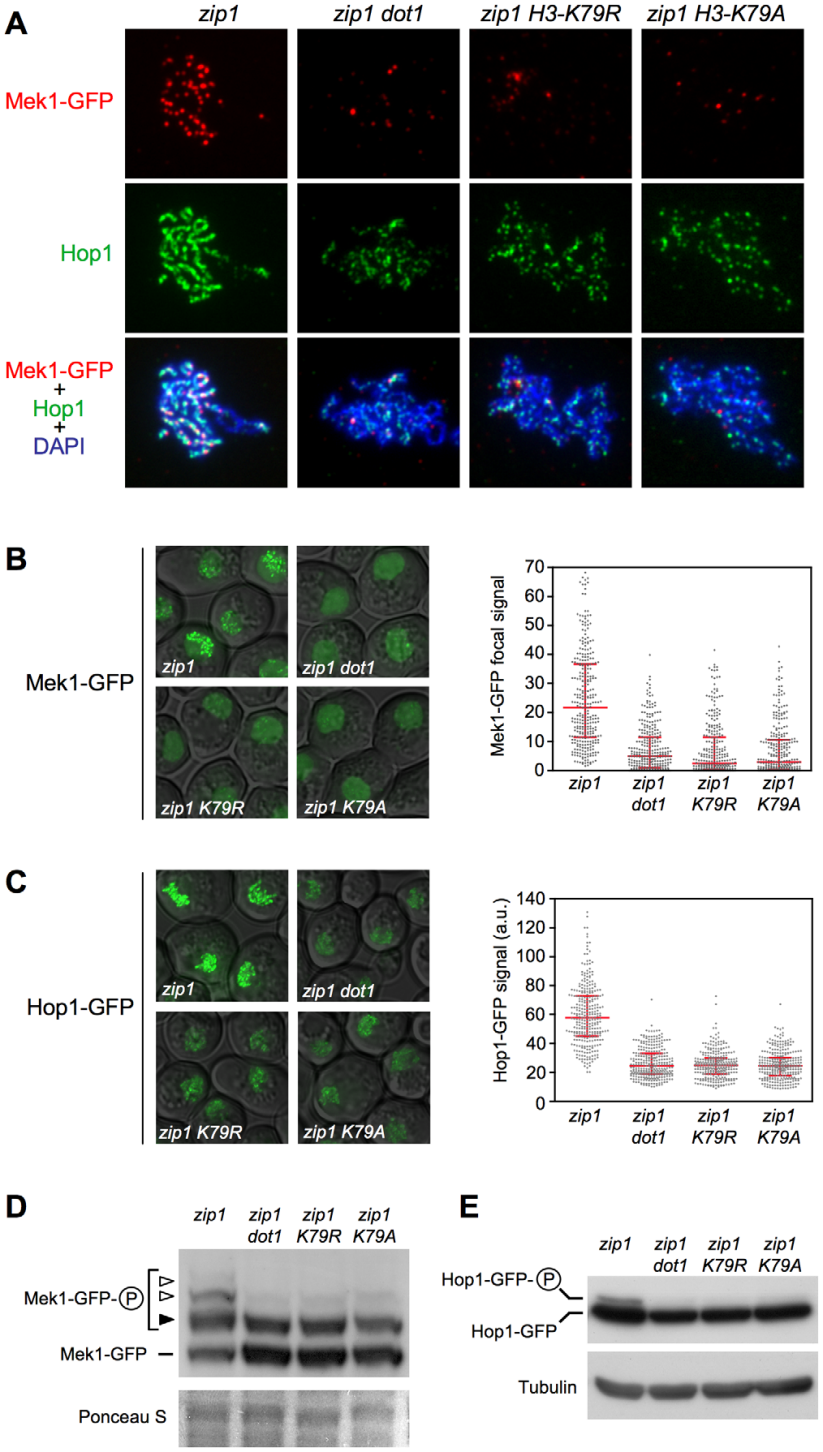
The *H3-K79R* and *H3-K79A* mutations recapitulate *dot1* defects in checkpoint-induced Mek1 and Hop1 phosphorylation and localization. (A) Immunofluorescence of meiotic chromosome spreads stained with DAPI (blue), anti-Hop1 (green) and anti-GFP (red) antibodies. Representative nuclei are shown. The same exposure time was used to capture the signal from the different strains. (B) and (C) Representative images of meiotic cells expressing *MEK1-GFP* and *HOP1-GFP*, respectively. The scattered plots represent the quantification of the Mek1-GFP focal signal (B) and total Hop1-GFP signal intensity (C) on fluorescence images (a.u., arbitrary units). Error bars represent the median with interquartile range. 300 individual nuclei were analyzed for each strain. (D) Western blot analysis of *zip1*-induced Mek1-GFP phosphorylation in a Phos-tag gel using anti-Mek1 antibodies. The basal Mek1-GFP form (line), and the forms resulting from Mec1/Tel1-dependent phosphorylation (black arrowhead) and autophosphorylation (white arrowheads) are indicated. Ponceau S staining of the membrane is shown as a loading control. (E) Western blot analysis of *zip1*-induced Hop1-GFP phosphorylation using anti-GFP antibodies. Tubulin is shown as a loading control. Strains in (A), (B) and (D) are: DP1046 (*zip1*), DP1049 (*zip1 dot1*), DP1048 (*zip1 H3-K79R*) and DP1047 (*zip1 H3-K79A*). Strains in (C) and (E) are: DP1042 (*zip1*), DP1045 (*zip1 dot1*), DP1044 (*zip1 H3-K79R*) and DP1043 (*zip1 H3-K79A*). In all cases (A–E), spreads were made, GFP images were captured and cell extracts were prepared after 24 h of meiotic induction in *ndt80* strains.

We have shown that, like *dot1*, mutation of H3K79 to non-methylatable residues completely bypasses the checkpoint-induced meiotic delay of *zip1* (Figure 1B). On the other hand, we have revealed that, in *zip1* cells, Dot1 orchestrates Hop1 and Mek1 activation and chromosomal distribution (Figure 2, Figure 3, Figure 4, and Figure 5). To confirm that Hop1 and Mek1 checkpoint functions are also directly regulated by H3K79me, and not by another possible methyltransferase-independent function of Dot1, we examined their phosphorylation and localization in the *zip1 H3-K79R* and *zip1 H3-K79A* mutants. We found that, indeed, these histone point mutants phenocopy the *dot1* defects in Mek1 foci formation (Figure 6A and 6B) and Mek1 autophosphorylation (Figure 6D; Figure S4). Likewise, the *zip1 H3-K79R* and *zip1 H3-K79A* mutants resemble *dot1* in the impaired Hop1 chromosomal distribution (Figure 6A and 6C; Figure S5) and checkpoint-induced phosphorylation (Figure 6E; Figure S4).

Thus, taken together, our results indicate that, upon meiotic recombination checkpoint triggering, Dot1-dependent H3K79 methylation promotes proper chromosomal localization and activation of Hop1, which in turn, is required to sustain Mek1 autophosphorylation and the ensuing checkpoint response.

### H3K79me partially controls Hop1 chromosomal localization via Pch2

Previous studies have shown that whereas in the *zip1* mutant the Pch2 meiotic checkpoint protein is detected only in the nucleolar (rDNA) region, in the *zip1 dot1* double mutant Pch2 is distributed throughout all chromatin [26]. To confirm that the regulation of Pch2 localization by Dot1 depends on the histone H3 methyltransferase activity, we analyzed Pch2 distribution on spread meiotic chromosomes of the *zip1 H3-K79R* and *zip1 H3-K79A* mutants. Although global Pch2 protein levels remained fairly invariable in the different mutants (Figure S4), we found that, like in *zip1 dot1*, Pch2 mislocalized to chromatin outside the rDNA in *zip1 H3-K79R* and *zip1 H3-K79A* strains (Figure 7A), suggesting that H3K79me excludes Pch2 from chromosomes.

Several lines of evidence support a role for Pch2 in promoting the turnover of Hop1 from meiotic chromosomes, at least in unperturbed meiosis [29], [39], [40]; therefore, it was possible that the reduced localization of Hop1 in the absence of Dot1 could stem from the action of the Pch2 protein aberrantly present at chromosomal locations removing Hop1 from *zip1* chromosomes. To investigate this possibility, we monitored Hop1 localization in *zip1 dot1 pch2* strains. Interestingly, we found that deletion of *PCH2* alleviated to some extent the defective Hop1 localization pattern of *zip1 dot1*, although it did not fully restore the high and continuous Hop1 levels present in *zip1* (Figure 7B–7D). To determine whether the increased abundance of Hop1 along chromosomes in *zip1 dot1 pch2* restores the checkpoint-induced delay we analyzed meiotic divisions and Mek1 phosphorylation (Figure 7E–7F). We found that the checkpoint was still impaired in the *zip1 dot1 pch2* triple mutant because, like the *zip1 dot1* and the *zip1 pch2* double mutants, it displayed wild-type kinetics of meiotic progression (Figure 7E) and defective Mek1 activation (Figure 7F), implying a more complex contribution of Pch2's function to the pachytene checkpoint response (see Discussion).

**Figure 8:**
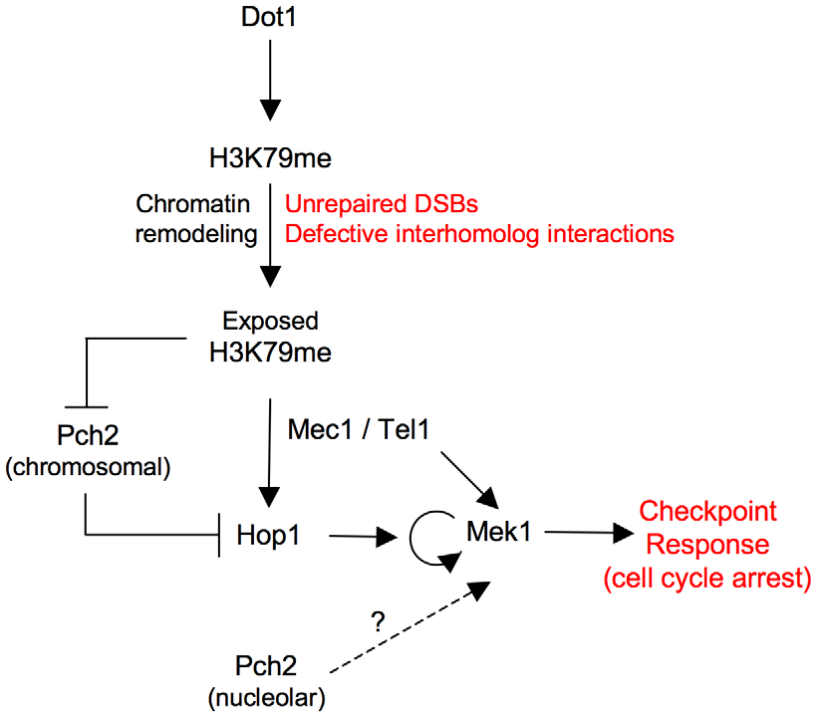
Model for Dot1 function in the meiotic recombination checkpoint. See text for details.

In summary, these observations indicate that in the *zip1* mutant, methylation of H3K79 by Dot1 controls proper chromosomal distribution of Hop1 by maintaining Pch2 confined in the nucleolar region. The fact that Hop1 localization is still partially impaired in the *zip1 dot1 pch2* triple mutant suggests that Dot1 may also regulate Hop1 chromosomal recruitment by a Pch2-independent mechanism (Figure 8).

## Discussion

Previous studies have shown that Dot1 is important for the pachytene checkpoint, but the molecular mechanism underlying such function remained unclear. Here, we provide evidence that methylation of H3K79 by Dot1 contributes to the meiotic recombination checkpoint response by enabling proper Hop1 chromosomal recruitment, which, in turn is a requisite for Mek1 activation by autophosphorylation.

We demonstrate that the function of Dot1 in the meiotic recombination checkpoint specifically relies on the methylation of H3K79, since the non-methylatable *H3-K79A* and *H3-K79R* mutations confer essentially the same meiotic phenotypes as the lack of Dot1. Moreover, by modulating Dot1 catalytic activity, we found that high levels of the H3K79-me3 are required for full checkpoint activation raising the possibility that this methylation state is particularly critical for promoting the proper localization of the Hop1 meiotic checkpoint adaptor (see below).

In mitotic cells, methylated histones are well-known chromatin marks for recognition of DSBs by checkpoint adaptors. In *S. cerevisiae*, the Rad9 adaptor is recruited to DSB sites by H3K79me [41], [42], whereas in *S. pombe*, which lacks H3K79me, the recruitment of the Crb2 adaptor relies on H4K20me [43]. In mammalian cells, the Rad9 and Crb2 homolog 53BP1 appears to recognize both H3K79me and H4K20me [44]–[46]. All these DNA damage checkpoint adaptors (Rad9, Crb2 and 53BP1) contain tandem tudor domains that mediate the interaction with the methylated histones. Rad9, Crb2 and 53BP1 also possess BRCT motifs; in fact, the recognition of DSBs by Rad9 and Crb2 in *S. cerevisiae* and *S. pombe*, respectively, is also mediated by their binding to phosphorylated histone H2A (hereafter γH2AX) via the BRCT domains [47], [48]. However, the Hop1 meiotic checkpoint adaptor lacks either tudor or BRCT motifs and contains a HORMA domain likely involved in protein-protein interactions [49], raising the possibility that its chromosomal recruitment can be mediated by different mechanisms.

As mentioned before, in DNA damaged vegetative cells, Rad9 function depends both on H3K79me and γH2AX [47], [50]–[52]; however, the relevance of both histone modifications appears to be different in meiotic cells. Dot1-dependent H3K79me is crucial for checkpoint function, at least in Zip1-deficient cells, because deletion of *DOT1* (or mutation of H3K79) results in complete bypass of the *zip1* meiotic block. In contrast, an H2A-S129* mutant, lacking the four C-terminal amino acids of histone H2A including the SQ phosphorylation site [53], has no defect in the *zip1*-induced checkpoint (Figure S6A). Moreover, like in both single mutants, meiotic progression and spore viability are essentially normal in the dot1 H2A-S129* double mutant (Figure S6A, S6B).

We show here that Dot1 is required for Mek1 and Hop1 activation in meiotically-challenged cells, but in addition to the checkpoint function, Mek1 and Hop1 promote the repair of meiotic DSBs by Dmc1-dependent interhomolog recombination [34], [37], [38], [54]. Consistent with this function, in the absence of Dmc1, Dot1 prevents the repair of DSBs by Rad54-dependent sister-chromatid recombination, which is controlled, at least in part, by inhibitory phosphorylation of Rad54 by Mek1 [26], [54]. In principle, it could be possible that impaired Hop1/Mek1 function in the absence of Dot1 could induce an alternative intersister recombination pathway resulting in meiotic progression because of the disappearance of the meiotic defects initially triggering the checkpoint. However, deletion of *DOT1* alleviates the meiotic arrest of *zip1 rad54* and *dmc1 rad54* mutants, where intersister repair is impaired, strongly suggesting that Dot1 performs a bona-fide meiotic checkpoint function [26]. The fact that, unlike Mek1 and Hop1, the Dot1 protein is dispensable in otherwise unperturbed meiosis implies the H3K79me is mostly relevant to signal defects when meiotic chromosome metabolism is disturbed (i.e., *zip1* or *dmc1* mutants). Consistent with this notion, Hop1 localization on *zip1* chromosomes is dramatically altered in the absence of Dot1, but it is only slightly reduced in the *dot1* single mutant as compared with the wild type (Figure 7C and 7D).

In other studies, activation of the Mek1 effector meiotic kinase has been monitored either by a slight electrophoretic mobility shift [32], [34] or by using an anti-phospho-Ser/Thr Akt substrate antibody, which specifically recognizes phosphorylation of Mek1 at T327 [33], [37], [55]. However, those assays do not permit one to delineate the different events contributing to Mek1 activation. Here, by using high-resolution Phos-tag gels, we identify several phosphorylated Mek1 forms and dissect the genetic requirements for sequential Mek1 activation. Our findings support a model (Figure 3E; Figure 8) in which the presence of unrepaired DSBs and/or unsynapsed chromosomes results in the initial phosphorylation of Mek1 by the redundant action of Mec1/Tel1. This priming phosphorylation is followed by autophosphorylation of Mek1 at T327 and T331 leading to full Mek1 activation supporting the checkpoint response. We found that Dot1 is chiefly required for this last step, which is mediated by Mek1 dimerization promoted by the Hop1 C-terminal domain [38]. Thus, the altered localization of Hop1 on *zip1 dot1* chromosomes likely explains the defect in Mek1 autophosphorylation. Interestingly, GST-mediated forced dimerization of Mek1 bypasses Dot1 requirement for its activation; however, this activation is only transient in the absence of Dot1, suggesting that proper chromosome axis architecture is required for maintenance of Mek1 activity.

We found that global levels of H3K79me do not significantly change in response to the meiotic defects of the *zip1* mutant, but this methylation is critical for the checkpoint response. The nature of the signal that triggers the meiotic checkpoint in *zip1* is still unclear. Like in mammals [56], the existence of a synapsis checkpoint in yeast has also been proposed [7], [55], [58]. Nevertheless, Dot1 is also required for the meiotic cell cycle arrest of the *dmc1* mutant that accumulates unrepaired DSBs [26], indicating that H3K79me is also involved in the response to meiotic DSBs. It has been reported that, under certain conditions, DSBs are efficiently repaired in *zip1* mutants [37] implying that the signal triggering the checkpoint could be different. However, Ddc2 foci marking the presence of recombination intermediates are detected in *zip1* [11] (Figure 2A), consistent with at least some DSBs remaining unrepaired in *zip1* mutants [57], [59], [60] sufficient to induce the checkpoint. Alternatively, or in addition, Mec1-Ddc2 may also sense defects in structural aspects of interhomolog interactions resulting from the lack of the central region of the SC [39]. In any case, independently of the nature of the signal triggering the meiotic checkpoint response(s), the question of how a constitutive histone mark, such as H3K79me, contributes to Hop1-mediated Mek1 activation specifically in challenged meiosis remains to be elucidated. In the DNA damage response in vegetative yeast cells or somatic mammalian cells it has been proposed, though never proven, that chromatin remodeling in the vicinity of DNA lesions may locally expose constitutive marks (i.e., H3K79me, H4K20me) supporting the recruitment of DNA damage checkpoint adaptors to activate the checkpoint [44], [45]. In meiotic cells, the DSB metabolism is linked to the special architecture of the chromosome axis [61]. Therefore, we envision that unrepaired DSBs and/or defects in interhomolog connections may provoke chromatin conformational changes unmasking H3K79me capable to drive proper Hop1 distribution along the axes, enabling its activation by Mec1 to elicit the downstream checkpoint events including Mek1 full activation by autophosphorylation (Figure 8).

Although it is formally possible that H3K79me may directly facilitate Hop1 recruitment to some extent, we provide evidence indicating that the control of Hop1 chromosomal distribution by H3K79me is substantially driven by regulation of the Pch2 protein. Pch2 was initially discovered as a meiotic checkpoint protein required for the *zip1*-induced meiotic arrest [29], but more recent studies have shown that Pch2 impacts multiple aspects of meiotic chromosome dynamics [55], [62]–[64]. In particular, Pch2 acts as a negative regulator of Hop1 chromosomal abundance [39], [40]. In wild-type pachytene chromosomes, Pch2 localizes to the unsynapsed rDNA region (nucleolus) and also along synapsed chromosomes [29], [40]. In contrast, Pch2 is solely detectable at the nucleolar region in the *zip1* mutant [29]; remarkably, in the absence of H3K79me, Pch2 is redistributed throughout all chromatin of *zip1* nuclei (Figure 7A). We hypothesize that, as a consequence of the synapsis defects of *zip1*, the H3K79me mark becomes exposed functioning as an anti-binding signal for Pch2, thus permitting the extensive Hop1 distribution found on *zip1* chromosomes (Figure 8). In the absence of Dot1 (or H3K79me), the presence of chromosomal Pch2 triggers the removal of Hop1 and the consequent defect in Mek1 activation. The reduced global levels of Hop1 detected in *zip1 dot1* (Figure S3 and Figure 6E) are also consistent with a higher protein turnover.

Interestingly, like in *zip1 dot1*, the synapsis checkpoint is still completely defective in the *zip1 dot1 pch2* triple mutant, despite the partial restoration of Hop1 localization. Since the excess of Hop1 induced by other means, such as *HOP1* overexpression, but in the presence of Pch2, does confer a meiotic delay in *zip1 dot1* and restores Mek1 phosphorylation (Figure 5), it is conceivable that nucleolar Pch2 performs an additional downstream function in Mek1 activation (Figure 8) and/or that the excess of Hop1 in the absence of Pch2 is not correctly assembled on chromosome axes to support checkpoint activation. In fact, the *zip1 pch2* mutant itself is also checkpoint deficient. Future studies will address these intriguing possibilities.

Dot1/DOT1L is structurally conserved throughout evolution from budding yeast to worms, flies, mice and humans; therefore, it is possible that members of the Dot1 family play similar roles in Metazoa. DOT1L is essential in mammals [65] functioning in embryogenesis, hematopoiesis and cardiac development [27]; however, much less is known about the impact of mammalian DOT1L in the DNA damage response. It would be interesting to determine whether, like the yeast counterpart, Dot1 orthologs are involved in meiotic checkpoint control in higher eukaryotes.

## Materials and Methods

### Yeast strains and plasmids

Yeast strains genotypes are listed in Table 1. All the strains are in the BR1919 background [66]. Gene deletions were made using a PCR-based approach [67], [68] except for *dot1::URA3*, *zip1::LYS2* and *ndt80::LEU2*, which were previously described [19], [26], [29]. *MEK1-13myc*, *MEK1-GFP* and *HOP1-GFP* were made by a PCR approach [68]. The C-terminally tagged Mek1-13myc and Mek1-GFP proteins are functional because spore viability of homozygous tagged wild-type diploids was similar to that of untagged strains and, in addition, they supported the checkpoint-induced delay of a *hop2* mutant. In *zip1 HOP1-GFP* strains the meiotic block was less tight, but Hop1-GFP displayed a localization pattern indistinguishable from that of the untagged protein (Figure S5); therefore, we used the native GFP fluorescence for quantitation of Hop1 localization. N-terminal tagging of Pch2 with three copies of the HA epitope has been previously described [29]. Strains carrying *DOT1* or *dot1-G401A* at its genomic locus or in the pRS315 vector (plasmids pRS315-DOT1 and pFvL54, respectively) were described [28], [31]. The *H3-K79A* and *H3-K79R* strains are deleted for all genomic copies of the histone H3-H4 encoding genes (*HHT1-HHF1 and HHT2-HHF2*) and express different versions of H3 from centromeric plasmids carrying either the *hht2(K79A)-HHF2* or *hht2(K79R)-HHF2* mutant genes (pFvL87 and pFvL88, respectively). The *mek1-T327A*, *mek1-T331A*, *mek1-K199R* mutations, as well as the *GST-MEK1* construct were introduced as described [33], using plasmids kindly provided by N. Hollingsworth (Stony Brook University, NY). The high-copy *HOP1* plasmid was also described [69]. Strains harboring the hta1-S129* and hta2-S129* mutations lacking the last four amino acids of the C-terminal tail of histone H2A including the serine 129 phosphorylated by Mec1/Tel1 [53] were made using plasmids pJHA16 and pJHA17 (provided by J. Downs, University of Sussex) following a pop-in/pop-out strategy. For meiotic time courses, strains were grown in 2×SC (3.5 ml) for 20–24 hours, then transferred to YPDA (2.5 ml) and incubated to saturation for additional 8 hours. Cells were harvested, washed with 2% potassium acetate (KAc), resuspended into 2% KAc (10 ml) and incubated at 30°C with vigorous shaking to induce meiosis and sporulation. Both YPDA and 2% KAc were supplemented with 20 mM adenine and 10 mM uracil. The culture volumes were scaled-up when needed.

**Table 1:** *Saccharomyces cerevisiae* strains.

Strain	Genotype*
BR1919-2N	*MAT* **a**/*MAT*α *leu2-3,112 his4-260 ura3-1 ade2-1 thr1-4 trp1-289*
DP409	BR1919-2N *zip1::LEU2*
DP419	BR1919-2N *hta1-S129* * *hta2-S129* *
DP420	BR1919-2N *hta1-S129* * *hta2-S129* * *zip1::LEU2*
DP421	BR1919-2N *lys2ΔNheI*
DP422	DP421 *zip1::LYS2*
DP424	DP421 *ndt80::LEU2*
DP428	DP421 *zip1::LYS2 ndt80::LEU2*
DP460	DP421 *zip1::LYS2 ndt80::LEU2 DDC2-GFP::TRP1*
DP555	DP421 *zip1::LYS2 dot1::kanMX6*
DP556	DP421 *zip1::LYS2 dot1::kanMX6::DOT1::URA3*
DP560	DP421 *zip1::LYS2 dot1::kanMX6::dot1-G401A::URA3*
DP579	DP421 *zip1::LYS2 ndt80::LEU2 dot1::URA3 DDC2-GFP::TRP1*
DP582	DP421 *zip1::LYS2 ndt80::LEU2 MEK1-GFP::kanMX6*
DP583	DP421 *zip1::LYS2 ndt80::LEU2 MEK1-GFP::kanMX6 dot1::URA3*
DP622	BR1919-2N *hta1-S129* * *hta2-S129* * *dot1::kanMX6*
DP623	BR1919-2N *hta1-S129* * *hta2-S129* * *dot1::kanMX6 zip1::LEU2*
DP624	DP421 *dot1::URA3*
DP625	DP421 *dot1::kanMX6*
DP655	DP421 *zip1::LYS2 ndt80::LEU2 dot1::kanMX6*
DP674	DP421 *zip1::LYS2 ndt80::LEU2 mek1::kanMX6*
DP680	DP421 *zip1::LYS2 ndt80::LEU2 sml1::kanMX6 mec1::KlURA3*
DP701	DP421 *zip1::LYS2 ndt80::LEU2 hop1::hphMX4*
DP713	DP421 *mek1::kanMX6*
DP714	DP421 *zip1::LYS2 mek1::kanMX6*
DP716	DP421 *zip1::LYS2 mek1::kanMX6 dot1::hphMX4*
DP728	BR1919-2N *zip1::kanMX6 ndt80::LEU2 spo11::hphMX4*
DP783	DP421 *zip1::LYS2 mek1::kanMX6 dot1::hphMX4 GST-mek1-K199R::URA3*
DP784	DP421 *zip1::LYS2 mek1::kanMX6 dot1::hphMX4 GST-mek1-T327A::URA3*
DP785	DP421 *zip1::LYS2 mek1::kanMX6 dot1::hphMX4 GST-MEK1::URA3*
DP790	DP421 *zip1::LYS2 mek1::kanMX6 GST-mek1-K199R::URA3*
DP791	DP421 *zip1::LYS2 mek1::kanMX6 GST-mek1-T327A::URA3*
DP792	DP421 *zip1::LYS2 mek1::kanMX6 GST-MEK1::URA3*
DP806	DP421 (*hht1-hhf1)::kanMX6 (hht2-hhf2)::natMX4 p[HHT2-HHF2]::TRP1*
DP807	DP421 (*hht1-hhf1)::kanMX6 (hht2-hhf2)::natMX4 p[hht2-K79A-HHF2]::TRP1*
DP808	DP421 (*hht1-hhf1)::kanMX6 (hht2-hhf2)::natMX4 p[hht2-K79R-HHF2]::TRP1*
DP809	DP421 (*hht1-hhf1)::kanMX6 (hht2-hhf2)::natMX4 p[HHT2-HHF2]::TRP1 zip1::LYS2*
DP810	DP421 (*hht1-hhf1)::kanMX6 (hht2-hhf2)::natMX4 p[hht2-K79A-HHF2]::TRP1 zip1::LYS2*
DP811	DP421 (*hht1-hhf1)::kanMX6 (hht2-hhf2)::natMX4 p[hht2-K79R-HHF2]::TRP1 zip1::LYS2*
DP812	DP421 (*hht1-hhf1)::kanMX6 (hht2-hhf2)::natMX4 p[HHT2-HHF2]::TRP1 zip1::LYS2 dot1:hphMX4*
DP848	DP421 *zip1::LYS2 ndt80::LEU2 MEK1-13myc::kanMX6*
DP849	DP421 *zip1::LYS2 ndt80::LEU2 MEK1-13myc::kanMX6 dot1::URA3*
DP861	DP421 *zip1::LYS2 ndt80::LEU2 sml1::kanMX6 mec1::KlURA3 tel1::hphMX4*
DP877	DP421 *zip1::LYS2 ndt80::LEU2 rad24::TRP1 tel1::hphMX4*
DP881	DP421 *zip1::LYS2 ndt80::LEU2 pch2::TRP1*
DP883	DP421 *zip1::LYS2 ndt80::LEU2 rad24::TRP1*
DP884	DP421 *zip1::LYS2 ndt80::LEU2 dot1::hphMX4 MEK1-13myc::kanMX6*
DP885	DP421 *zip1::LYS2 ndt80::LEU2 mek1::kanMX6 MEK1::URA3*
DP886	DP421 *zip1::LYS2 ndt80::LEU2 mek1::kanMX6 mek1-T327A::URA3*
DP887	DP421 *zip1::LYS2 ndt80::LEU2 mek1::kanMX6 mek1-T331A::URA3*
DP888	DP421 *zip1::LYS2 ndt80::LEU2 mek1::kanMX6 mek1-K199R::URA3*
DP890	DP421 *zip1::LYS2 ndt80::LEU2 mek1::kanMX6 MEK1::URA3 dot1::hphMX4*
DP963	DP421 *ndt80::LEU2 HOP1-GFP::kanMX6*
DP964	DP421 *zip1::LYS2 ndt80::LEU2 HOP1-GFP::kanMX6*
DP965	DP421 *zip1::LYS2 ndt80::LEU2 HOP1-GFP::kanMX6 dot1::URA3*
DP966	DP421 *ndt80::LEU2 HOP1-GFP::kanMX6 dot1::URA3*
DP1024	DP421 *zip1::LYS2 ndt80::LEU2 ddc2::TRP1 sml1::kanMX6*
DP1027	DP421 *zip1::LYS2 ndt80::LEU2 pch2::TRP1 dot1::URA3 HOP1-GFP::kanMX6*
DP1029	DP421 *zip1::LYS2 pch2::TRP1*
DP1041	DP421 *zip1::LYS2 pch2::TRP1 dot1::URA3*
DP1042	DP421 (*hht1-hhf1)::natMX4 (hht2-hhf2)::hphMX4 zip1::LYS2 ndt80::LEU2 HOP1-GFP::kanMX6 p[HHT2-HHF2]::TRP1*
DP1043	DP421 (*hht1-hhf1)::natMX4 (hht2-hhf2)::hphMX4 zip1::LYS2 ndt80::LEU2 HOP1-GFP::kanMX6 p[hht2-K79A-HHF2]::TRP1*
DP1044	DP421 (*hht1-hhf1)::natMX4 (hht2-hhf2)::hphMX4 zip1::LYS2 ndt80::LEU2 HOP1-GFP::kanMX6 p[hht2-K79R-HHF2]::TRP1*
DP1045	DP421 (*hht1-hhf1)::natMX4 (hht2-hhf2)::hphMX4 zip1::LYS2 ndt80::LEU2 dot1::URA3 HOP1-GFP::kanMX6 p[HHT2-HHF2]::TRP1*
DP1046	DP421 (*hht1-hhf1)::natMX4 (hht2-hhf2)::hphMX4 zip1::LYS2 ndt80::LEU2 MEK1-GFP::kanMX6 p[HHT2-HHF2]::TRP1*
DP1047	DP421 (*hht1-hhf1)::natMX4 (hht2-hhf2)::hphMX4 zip1::LYS2 ndt80::LEU2 MEK1-GFP::kanMX6 p[hht2-K79A-HHF2]::TRP1*
DP1048	DP421 (*hht1-hhf1)::natMX4 (hht2-hhf2)::hphMX4 zip1::LYS2 ndt80::LEU2 MEK1-GFP::kanMX6 p[hht2-K79R-HHF2]::TRP1*
DP1049	DP421 (*hht1-hhf1)::natMX4 (hht2-hhf2)::hphMX4 zip1::LYS2 ndt80::LEU2 dot1::URA3 MEK1-GFP::kanMX6 p[HHT2-HHF2]::TRP1*
DP1050	DP421 (*hht1-hhf1)::natMX4 (hht2-hhf2)::hphMX4 zip1::LEU2 PCH2-3HA p[HHT2-HHF2]::TRP1*
DP1051	DP421 (*hht1-hhf1)::natMX4 (hht2-hhf2)::hphMX4 zip1::LEU2 PCH2-3HA p[hht2-K79A-HHF2]::TRP1*
DP1052	DP421 (*hht1-hhf1)::natMX4 (hht2-hhf2)::hphMX4 zip1::LEU2 PCH2-3HA p[hht2-K79R-HHF2]::TRP1*
DP1053	DP421 (*hht1-hhf1)::natMX4 (hht2-hhf2)::hphMX4 zip1::LEU2 PCH2-3HA dot1::kanMX6 p[HHT2-HHF2]::TRP1*
DP1054	DP421 *zip1::LYS2 ndt80::LEU2 pch2::TRP1 dot1::URA3*

* All strains are isogenic diploids homozygous for the indicated markers.

### Western blotting and analysis of Mek1 phosphorylation

TCA cell extracts from 5–10 ml of sporulating cultures were processed as described [14]. To resolve the phosphorylated forms of Mek1 or Mek1-GFP, 10% or 7% gels (acrylamide∶bisacrylamide 29∶1), respectively, containing 37.5 µM Phos-tag (Wako Chemicals) and 75 µM MnCl_2_ were used. Gels were run on ice at 100 volts in a MiniProtean3 (Bio-Rad) for 3 h. After running, gels were washed with 1 mM EDTA before transfer to PVDF membranes.

For dephosphorylation assays, total TCA cell extracts solubilized in Laemmli buffer were diluted 10 times with phosphatase buffer supplemented with 1 mM MnCl_2_. Diluted extracts were treated with 2000 units of lambda phosphatase (New England Biolabs) for 30 min at 30°C. As control, a similar aliquot of the diluted extract was incubated under the same conditions but without adding phosphatase. Samples were re-precipitated with 20% TCA, washed with acetone, boiled in Laemmli buffer and loaded in Phostag gels.

The following antibodies were used: rabbit polyclonal anti-Mek1 (1∶1000 dilution) [11] and anti-Dot1 (1∶2000 dilution) [31]. Rabbit polyclonal anti-H3K79-me1 (ab2886; 1∶1000 dilution), anti-H3K79-me2 (ab3594; 1∶2000 dilution), anti-H3K79-me3 (ab2621; 1∶2000 dilution), and anti-histone H3 (ab1791: 1∶5000) were from Abcam. Rabbit polyclonal anti-Hop1 (1∶2000 dilution) [3], was from S. Roeder (Yale University). Anti-Cdc5 (sc-6733; 1∶1000 dilution) was from Santa Cruz Biotechnology. Mouse monoclonal anti-HA (12CA5; 1∶2000 dilution) was from Roche. Anti-phosphoglycerate kinase (PGK) (A-6457, 1∶10000 dilution) was from Molecular Probes. The ECL or ECL+ reagents were used for detection. The signal was captured on film and/or with a ChemiDoc XRS (Bio-Rad) system and quantified with the Quantity One software (Bio-Rad).

### Cytology

Immunofluorescence of chromosome spreads was performed essentially as described [29]. To detect Mek1-myc and Mek1-GFP, mouse monoclonal anti-myc (clone 4A6, Millipore) and mouse monoclonal anti-GFP (JL-8, Clontech) antibodies, respectively, were used at 1∶200 dilution. Rabbit polyclonal anti-Red1 and anti-Hop1 antibodies (gifts from S. Roeder) have been previously described [3], [5]. Anti-mouse and/or anti-rabbit AF-488 and AF-594 conjugated secondary antibodies (Molecular Probes) were used at 1∶200 dilution. Images were captured with a Nikon Eclipse 90i fluorescence microscope controlled with the MetaMorph software and equipped with an Orca-AG (Hammamatsu) CCD camera and a PlanApo VC 100×1.4 NA objective.

Whole cell images were captured with an Olympus IX71 fluorescence microscope equipped with a personal DeltaVision system (Applied Precision), a CoolSnap HQ2 (Photometrics) camera and a 100× UPLSAPO 1.4 NA objective. Exposure times were 800 ms, 400 ms and 300 ms for Ddc2-GFP, Mek1-GFP, and Hop1-GFP, respectively. Stacks of 20 planes at 0.2 µm intervals were captured. Maximum intensity projections of deconvolved images were generated with the SoftWoRx 5.0 software (Applied Precision). Quantification of GFP signals in the projections of individual nuclei was performed with the Image J software (http://rsb.info.nih.gov/ij/). Background signal was subtracted using the Otsu's or the Renyi's entropy threshold methods in Image J. To outline the contour of the cells in the representative whole-cell images presented, an overlay of the DIC image with 15–20% transparency over the GFP signal is shown.

### Other techniques

To analyze meiotic nuclear divisions, cells were fixed in 70% ethanol, washed in PBS and stained with 1 µg/µl DAPI for 15 minutes at room temperature. At least 300 cells of every strain were scored at each time point. Analyses of meiotic kinetics were repeated several times; representative time courses are shown. Spore viability was determined by tetrad dissection. To calculate the statistical significance of differences a two-tailed Student *t-*test was used. *P-*values were calculated using the GraphPad Prism 4.0 software. *P*<0.01 was considered significant.

## Supporting Information

Figure S1 H3K79 is constitutively methylated during meiosis. (A) Western blot analysis of H3K79 methylation dynamics throughout meiosis. Total histone H3 is shown as a loading control. Strains are: DP421 (wild type), DP625 (*dot1*), DP422 (*zip1*) and DP555 (*zip1 dot1*). (B) H3K79 methylation does not change in other mutants defective in the meiotic recombination checkpoint. Western blot analysis of H3K79me in *ndt80*-arrested cells at 24 h after meiosis induction. Total histone H3 is shown as a loading control. Strains are: DP428 (*zip1*), DP728 (*zip1 spo11*), DP881 (*zip1 pch2*), DP883 (*zip1 rad24*) and DP1024 (*zip1 ddc2*).
(TIF)

Figure S2 Red1 linear localization in *zip1* chromosomes is not significantly altered in the absence of Dot1. Immunofluorescence of meiotic chromosome spreads stained with DAPI (blue) and anti-Red1 (green) antibody. Representative nuclei are shown. Spreads were prepared 24 h after meiotic induction of *ndt80* cells. Strains are: DP848 (*zip1*) and DP849 (*zip1 dot1*).
(TIF)

Figure S3 Dot1 is required for *zip1*-induced Hop1 phosphorylation. Western blot analysis of Hop1 in cell extracts obtained 24 h after meiotic induction in *ndt80* cells. Ponceau S staining of the membrane was used a loading control. Strains are: DP428 (*zip1*), DP674 (*zip1 mek1*), DP655 (*zip1 dot1*), DP728 (*zip1 spo11*) and DP680 (*zip1 mec1*).
(TIF)

Figure S4 Pch2 protein levels do not change in the absence of H3K79me. Western blot analysis of Pch2-HA in cell extracts obtained 15 h after meiotic induction. PGK is shown as a loading control. Hop1 and Mek1 phosphorylation were also analyzed in the same samples to demonstrate their defective activation in the *H3-K79R* and *H3-K79A* mutants. See Figure 3 for explanation of the black and white arrowheads pointing to phosphorylated Mek1 forms. Strains are: DP1050 (*zip1*), DP1053 (*zip1 dot1*), DP1052 (*zip1 H3-K79R*) and DP1051 (*zip1 H3-K79A*).
(TIF)

Figure S5 Hop1-GFP localization is impaired in the absence of H3K79me. Immunofluorescence of meiotic chromosome spreads stained with DAPI (blue), anti-Red1 (green) and anti-GFP (red) antibodies. Representative nuclei are shown. Spreads were prepared 24 h after meiotic induction of *ndt80* cells. Strains are: DP1042 (*zip1*), DP1045 (*zip1 dot1*), DP1044 (*zip1 H3-K79R*) and DP1043 (*zip1 H3-K79A*).
(TIF)

Figure S6 Analysis of γH2AX meiotic function. (A) Unlike H3K79me, γH2AX is not required for the checkpoint-induced by *zip1* because the H2A-S129* mutation does not suppress *zip1* meiotic block. Time course of meiotic nuclear divisions; the percentage of cells containing more than two nuclei is represented. Strains are: BR1919-2N (wild type), DP409 (*zip1*), DP419 (H2A-S129*), DP420 (zip1 H2A-S129*), DP622 (dot1 H2A-S129*) and DP623 (zip1 dot1 H2A-S129*). (B) Spore viability is high in the absence of γH2AX and H3K79me, suggesting that both histone modifications are not required in unperturbed meiosis. At least 288 spores were scored for each strain. Means and standard deviations are shown. Strains are: BR1919-2N (wild type), DP419 (H2A-S129*), DP622 (dot1 H2A-S129*) and DP624 (*dot1*).
(TIF)

Video S1 Hop1 chromosomal distribution is impaired in the absence of Dot1. 3D reconstruction of deconvolved Z-stack images showing Hop1-GFP signal in *zip1* and *zip1 dot1* cells. Two different nuclei of each strain are shown during the movie.
(MOV)
