# Supplementary figures and images for: Dot1-Dependent Histone H3K79 Methylation Promotes Activation of the Mek1 Meiotic Checkpoint Effector Kinase by Regulating the Hop1 Adaptor
Source: PLoS Genet. 2013 Jan 31;9(1):e1003262. doi: 10.1371/journal.pgen.1003262 (PMC3561090; doi:10.1371/journal.pgen.1003262)

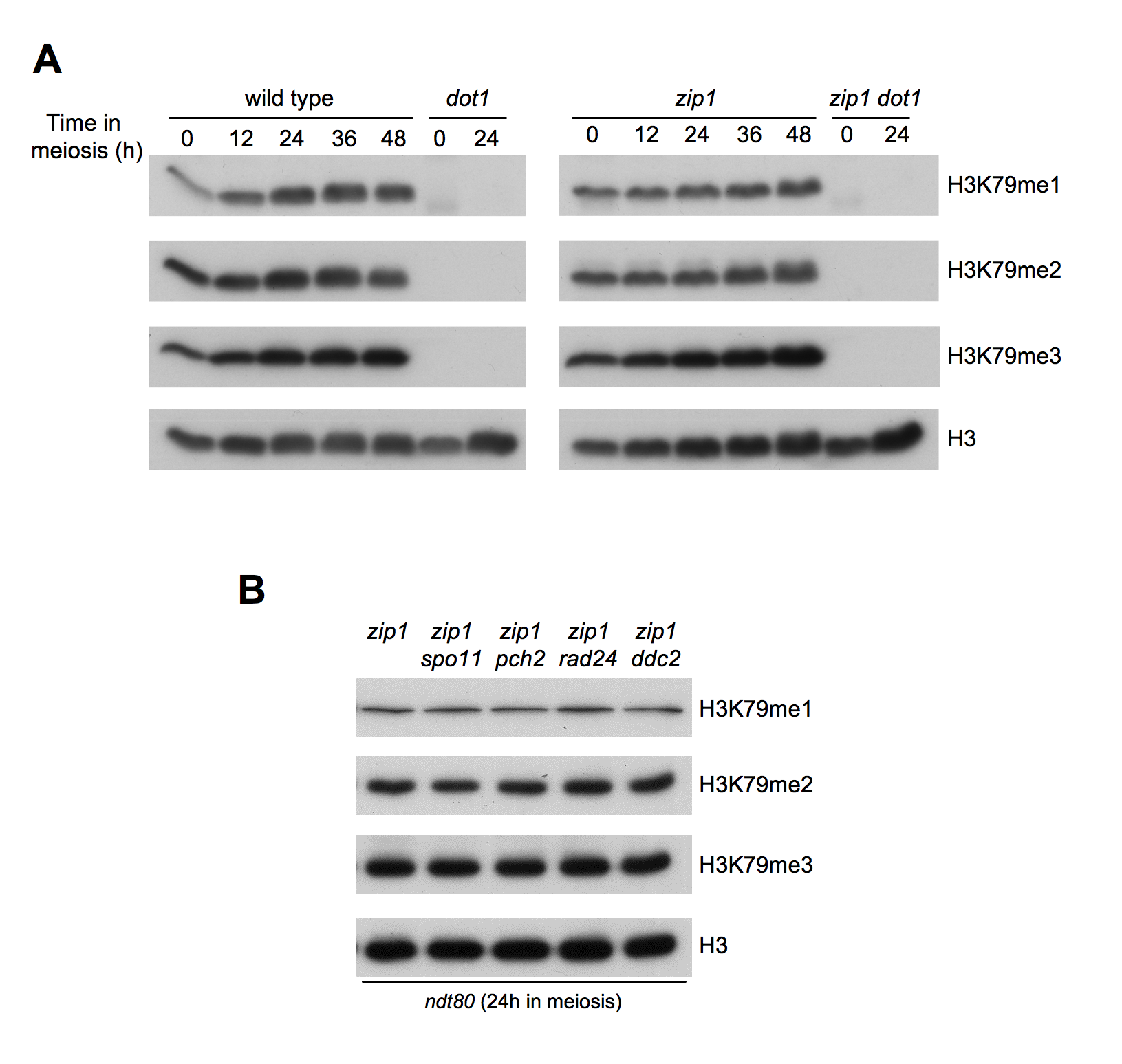

Supplement: Figure S1 — H3K79 is constitutively methylated during meiosis. (A) Western blot analysis of H3K79 methylation dynamics throughout meiosis. Total histone H3 is shown as a loading control. Strains are: DP421 (wild type), DP625 (dot1), DP422 (zip1) and DP555 (zip1 dot1). (B) H3K79 methylation does not change in other mutants defective in the meiotic recombination checkpoint. Western blot analysis of H3K79me in ndt80-arrested cells at 24 h after meiosis induction. Total histone H3 is shown as a loading control. Strains are: DP428 (zip1), DP728 (zip1 spo11), DP881 (zip1 pch2), DP883 (zip1 rad24) and DP1024 (zip1 ddc2). (TIF) [file pgen.1003262.s001.tif]

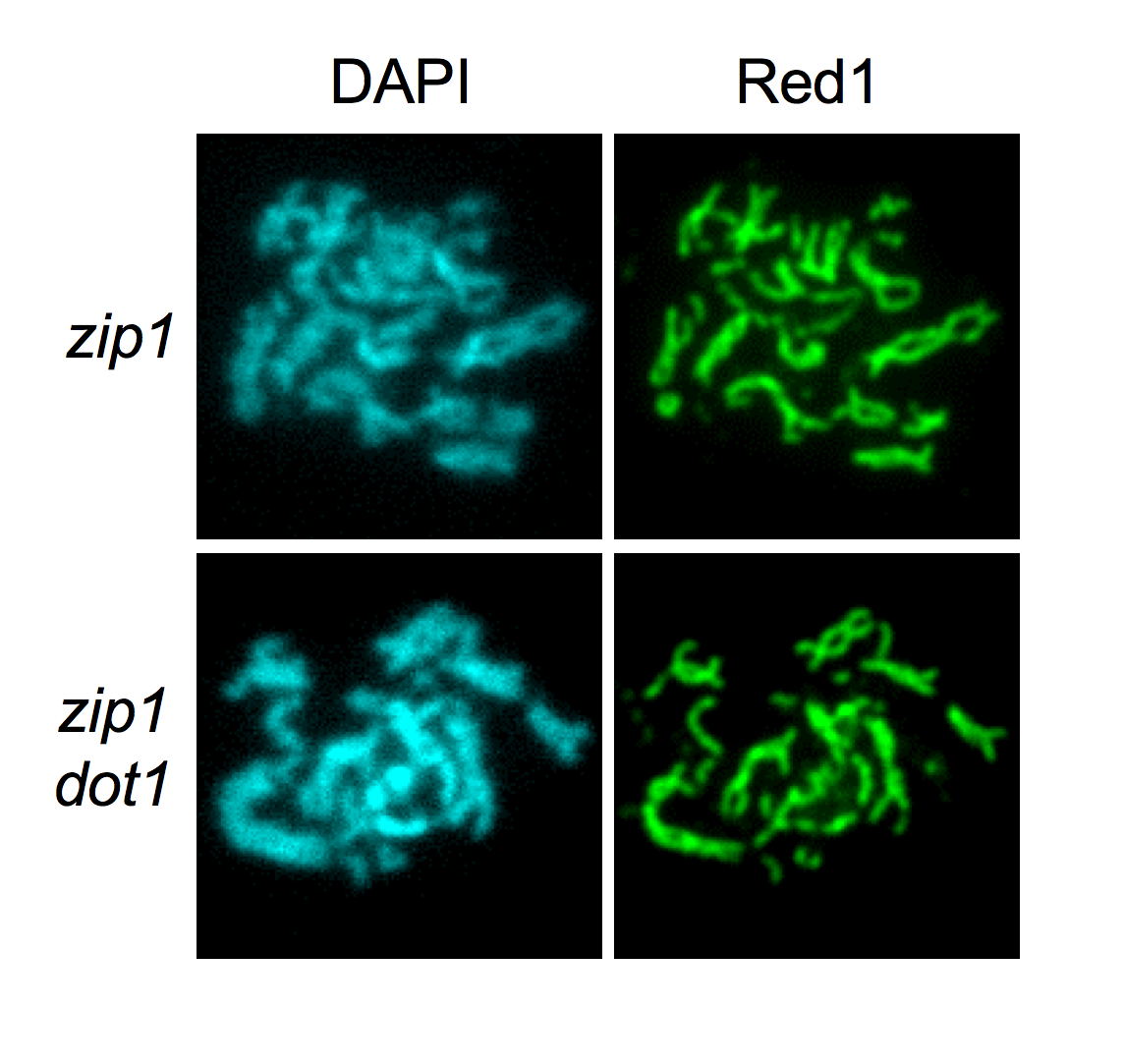

Supplement: Figure S2 — Red1 linear localization in zip1 chromosomes is not significantly altered in the absence of Dot1. Immunofluorescence of meiotic chromosome spreads stained with DAPI (blue) and anti-Red1 (green) antibody. Representative nuclei are shown. Spreads were prepared 24 h after meiotic induction of ndt80 cells. Strains are: DP848 (zip1) and DP849 (zip1 dot1). (TIF) [file pgen.1003262.s002.tif]

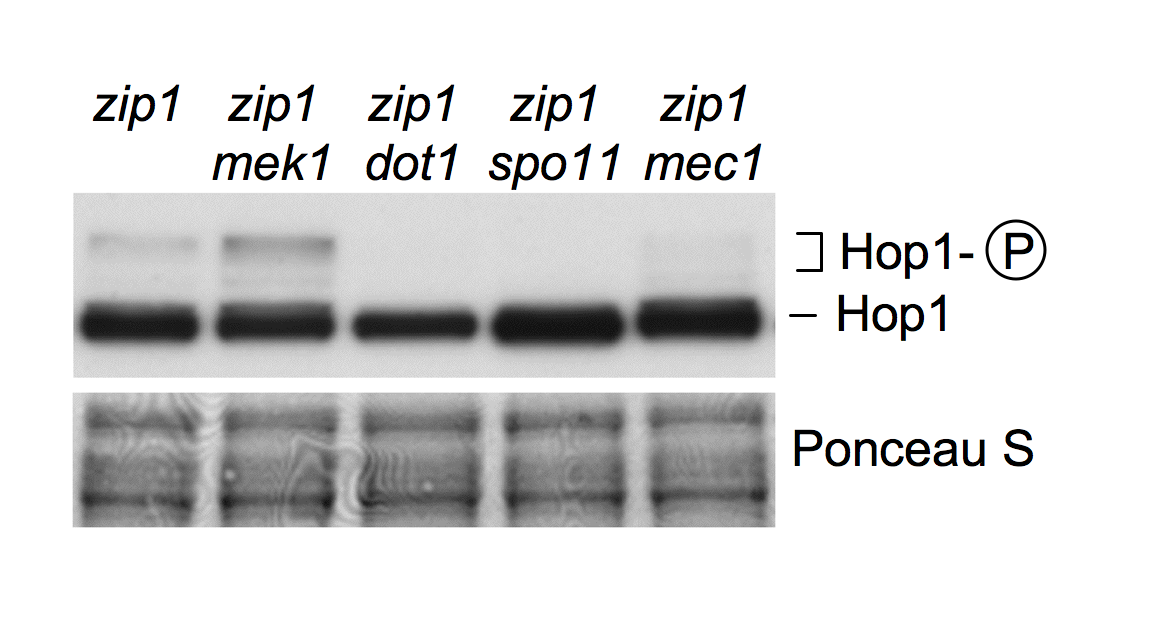

Supplement: Figure S3 — Dot1 is required for zip1-induced Hop1 phosphorylation. Western blot analysis of Hop1 in cell extracts obtained 24 h after meiotic induction in ndt80 cells. Ponceau S staining of the membrane was used a loading control. Strains are: DP428 (zip1), DP674 (zip1 mek1), DP655 (zip1 dot1), DP728 (zip1 spo11) and DP680 (zip1 mec1). (TIF) [file pgen.1003262.s003.tif]

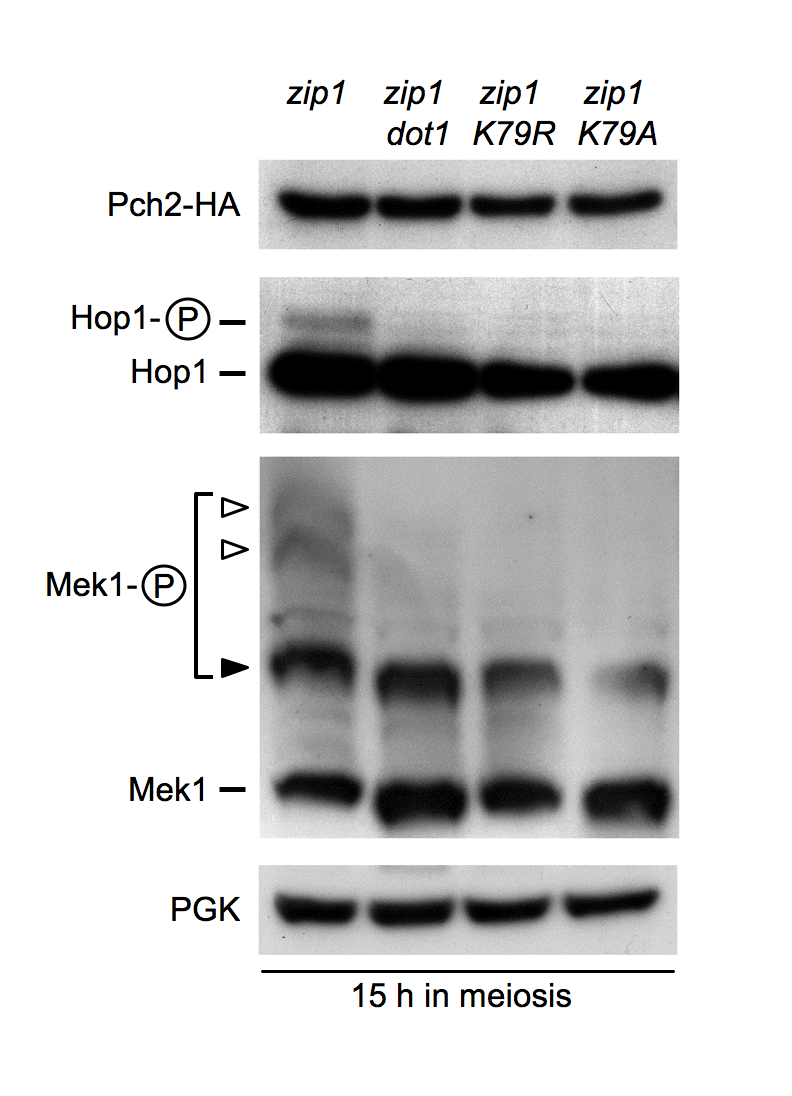

Supplement: Figure S4 — Pch2 protein levels do not change in the absence of H3K79me. Western blot analysis of Pch2-HA in cell extracts obtained 15 h after meiotic induction. PGK is shown as a loading control. Hop1 and Mek1 phosphorylation were also analyzed in the same samples to demonstrate their defective activation in the H3-K79R and H3-K79A mutants. See Figure 3 for explanation of the black and white arrowheads pointing to phosphorylated Mek1 forms. Strains are: DP1050 (zip1), DP1053 (zip1 dot1), DP1052 (zip1 H3-K79R) and DP1051 (zip1 H3-K79A). (TIF) [file pgen.1003262.s004.tif]

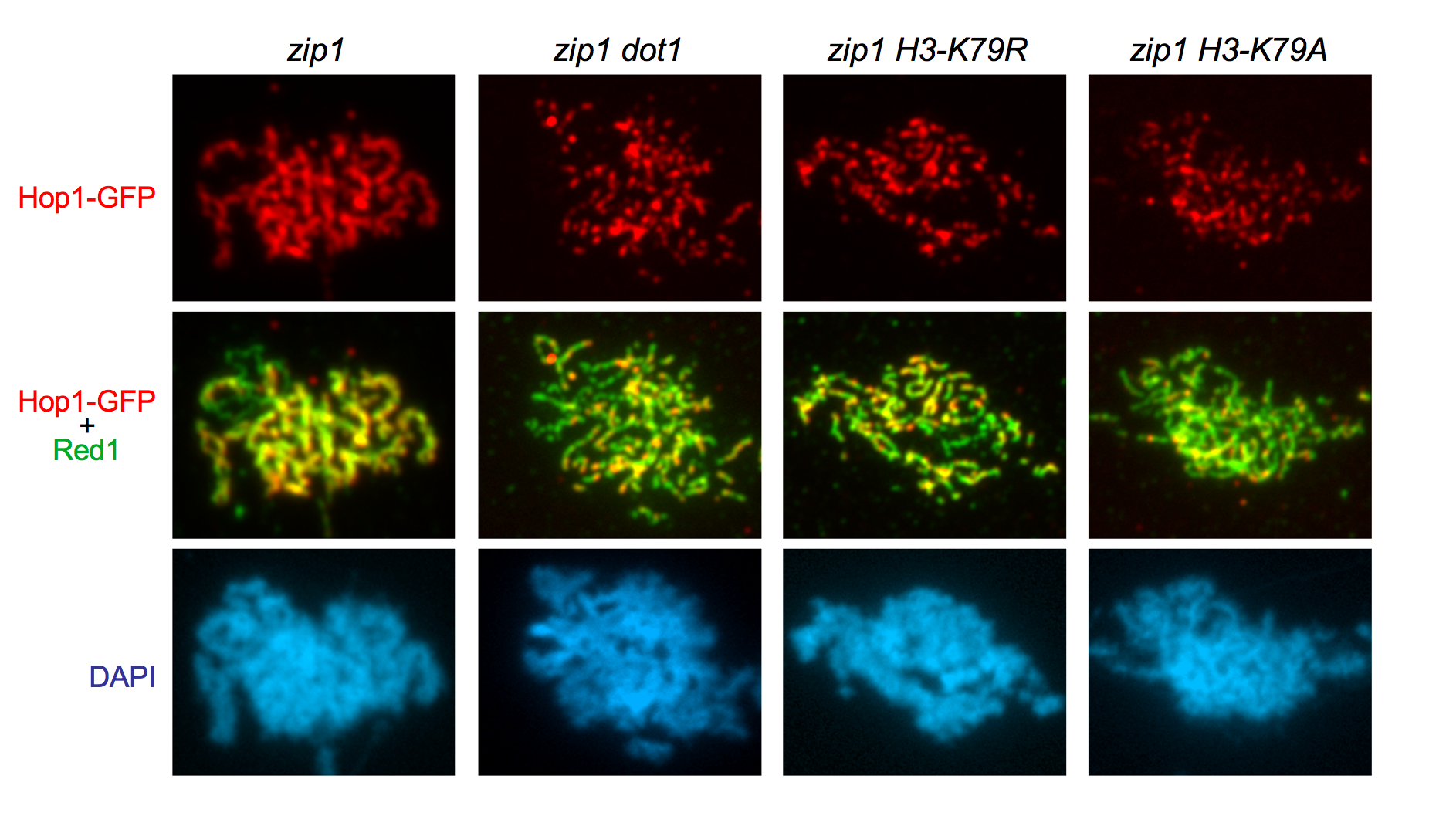

Supplement: Figure S5 — Hop1-GFP localization is impaired in the absence of H3K79me. Immunofluorescence of meiotic chromosome spreads stained with DAPI (blue), anti-Red1 (green) and anti-GFP (red) antibodies. Representative nuclei are shown. Spreads were prepared 24 h after meiotic induction of ndt80 cells. Strains are: DP1042 (zip1), DP1045 (zip1 dot1), DP1044 (zip1 H3-K79R) and DP1043 (zip1 H3-K79A). (TIF) [file pgen.1003262.s005.tif]

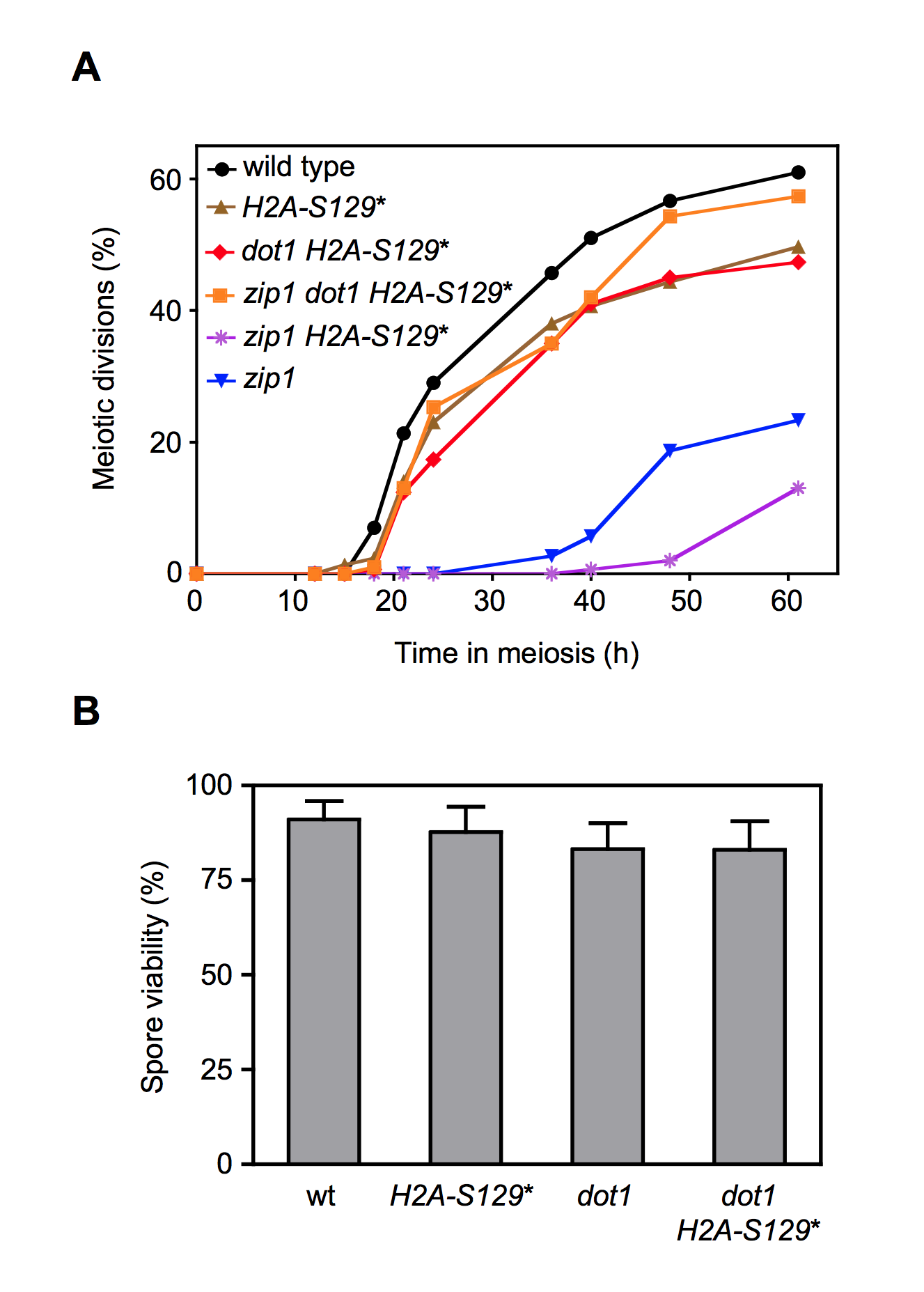

Supplement: Figure S6 — Analysis of γH2AX meiotic function. (A) Unlike H3K79me, γH2AX is not required for the checkpoint-induced by zip1 because the H2A-S129* mutation does not suppress zip1 meiotic block. Time course of meiotic nuclear divisions; the percentage of cells containing more than two nuclei is represented. Strains are: BR1919-2N (wild type), DP409 (zip1), DP419 (H2A-S129*), DP420 (zip1 H2A-S129*), DP622 (dot1 H2A-S129*) and DP623 (zip1 dot1 H2A-S129*). (B) Spore viability is high in the absence of γH2AX and H3K79me, suggesting that both histone modifications are not required in unperturbed meiosis. At least 288 spores were scored for each strain. Means and standard deviations are shown. Strains are: BR1919-2N (wild type), DP419 (H2A-S129*), DP622 (dot1 H2A-S129*) and DP624 (dot1). (TIF) [file pgen.1003262.s006.tif]
